# Inflammatory expression profiles in monocyte-to-macrophage differentiation in patients with systemic lupus erythematosus and relationship with atherosclerosis

**DOI:** 10.1186/ar4609

**Published:** 2014-07-10

**Authors:** Benjamin D Korman, Chiang-Ching Huang, Carly Skamra, Peggy Wu, Renee Koessler, David Yao, Qi Quan Huang, William Pearce, Kim Sutton-Tyrrell, George Kondos, Daniel Edmundowicz, Richard Pope, Rosalind Ramsey-Goldman

**Affiliations:** 1 Northwestern University Feinberg School of Medicine, 240 E Huron Street, McGaw M-230, Chicago, IL 60611, USA; 2 University of Pittsburgh, 132 Parran Hall, Pittsburgh, PA 15261, USA; 3 University of Illinois at Chicago, 840 S. Wood St., (MC 715), Chicago, IL 60612, USA; 4 Temple University, 610 University Services Bldg.1601 N. Broad St. Philadelphia, Philadelphia, PA 19122, USA

## Abstract

**Introduction:**

Our objectives were to examine mononuclear cell gene expression profiles in patients with systemic lupus erythematosus (SLE) and healthy controls and to compare subsets with and without atherosclerosis to determine which genes’ expression is related to atherosclerosis in SLE.

**Methods:**

Monocytes were obtained from 20 patients with SLE and 16 healthy controls and were *in vitro*-differentiated into macrophages. Subjects also underwent laboratory and imaging studies to evaluate for subclinical atherosclerosis. Whole-genome RNA expression microarray was performed, and gene expression was examined.

**Results:**

Gene expression profiling was used to identify gene signatures that differentiated patients from controls and individuals with and without atherosclerosis. In monocytes, 9 out of 20 patients with SLE had an interferon-inducible signature compared with 2 out of 16 controls. By looking at gene expression during monocyte-to-macrophage differentiation, we identified pathways which were differentially regulated between SLE and controls and identified signatures based on relevant intracellular signaling molecules which could differentiate SLE patients with atherosclerosis from controls. Among patients with SLE, we used a previously defined 344-gene atherosclerosis signature in monocyte-to-macrophage differentiation to identify patient subgroups with and without atherosclerosis. Interestingly, this signature further classified patients on the basis of the presence of SLE disease activity and cardiovascular risk factors.

**Conclusions:**

Many genes were differentially regulated during monocyte-to-macrophage differentiation in SLE patients compared with controls. The expression of these genes in mononuclear cells is important in the pathogenesis of SLE, and molecular profiling using gene expression can help stratify SLE patients who may be at risk for development of atherosclerosis.

## Introduction

Systemic lupus erythematosus (SLE) is a chronic multisystem autoimmune disease with a diverse array of clinical manifestations. More recently, it has become evident that SLE is associated with both accelerated atherosclerosis and an increased risk of cardiovascular complications. Women with SLE have a high prevalence of coronary heart disease, and in some cohorts this figure is as high as 6% to 10% [1-4]. The estimated incidence of new cardiovascular events in patients with SLE is approximately 1.2% to 1.5% per year, which represents a five- to six-fold age-adjusted increased risk compared with women without SLE, which is even more striking since it occurs in young pre-menopausal women [5]. Although chronic inflammation is thought to be an important risk factor for these manifestations and a number of cytokines (including interferons) have been proposed as important in this process, the exact pathogenesis of atherosclerosis in SLE remains to be elucidated [6].

Monocytes recruited into tissues from peripheral blood differentiate into macrophages, which are critical in the pathogenesis of many diseases, including both atherosclerosis and SLE [7]. Atherosclerosis has long been associated with chronic inflammation, and previous work has shown that monocytes and macrophages play an important role in the development of atherosclerosis [8,9]. Recent studies have shown that patients with SLE have monocyte/macrophage defects involving surface protein expression, cytokine production, and phagocytic capacity, suggesting that monocytes and macrophages are key players in the pathobiology of SLE [10].

RNA expression microarray has been previously used to analyze mononuclear cells from patients with SLE and has identified an interferon-inducible gene expression profile which is seen in a significant proportion of patients with SLE and tends to correlate with more severe disease manifestations [11-14]. Similar analyses have been done in patients with coronary artery disease and atherosclerosis and have found strong differential expression of genes related to innate and adaptive immunity, particularly those involved in leukocyte transendothelial migration [15-18].

There are dramatic changes in gene expression profiles during monocyte-to-macrophage differentiation, including genes important in lipid metabolism and foam cell function [19,20]. Furthermore, since monocytes differentiate into macrophages as they egress from the circulation into the inflammatory or atherosclerotic milieu, we reasoned that comparing this process in patients with SLE and controls might reveal differences that would provide insights into the increased risk for atherosclerosis seen in patients with SLE. To test this hypothesis, we examined gene expression profiles in monocytes and *in vitro*-differentiated macrophages in SLE patients and healthy controls with and without subclinical atherosclerosis to look for differential expression patterns that might help explain the increased coincidence of these diseases.

## Materials and methods

Peripheral blood was obtained from 36 females: 20 patients with SLE and 16 healthy controls. The institutional review boards of Northwestern University and the University of Illinois at Chicago approved the study, and all participants gave written informed consent prior to their involvement.

SLE patients and control subjects were selected from the SOLVABLE study (Study of SLE Vascular and Bone Long-term Endpoints, a prospective study assessing risk for subclinical and clinical cardiovascular disease) on the basis of having either evidence of atherosclerosis or no evidence of atherosclerosis. All patients had a physician-verified SLE diagnosis and met at least four American College of Rheumatology (ACR) criteria for the classification of SLE. Controls were selected from the general population and represent healthy women who were matched to patients with SLE by age (±5 years), race/ethnicity, and zip code of residence [21]. Controls did not have evidence of SLE or other autoimmune disease.

At study visits, subjects filled out a questionnaire in which they provided demographic, personal, and family history and information about cardiovascular risk factors and underwent routine physical examination and laboratory tests. Patient subphenotypes were determined on the basis of whether patients met each of the 11 ACR classification criteria. Information on age, self-reported race/ethnicity, smoking history, current estrogen use, current aspirin use, and menopause status was obtained from a questionnaire. (If indeterminate by questionnaire, menopause was determined by follicle-stimulating hormone status). Blood pressure was measured twice, and the mean of the two measurements was used for analysis. Height, weight, and waist/hip measurements were obtained by using the protocol in the Multi-ethnic Study of Atherosclerosis [22].

Laboratory tests were run on all patients and controls. These included fasting lipids (total cholesterol, high-density lipoprotein cholesterol (HDL-c), and triglyceride) and fasting glucose measured in the Lipid Laboratory at the University of Pittsburgh Graduate School of Public Health. The Friedewald equation was used to estimate low-density lipoprotein cholesterol (LDL-c) unless the triglyceride level was more than 400 mg/dL, in which case LDL-c was measured directly. Plasma glucose levels were determined by enzymatic assay, and plasma insulin levels were measured by radioimmunoassay. C-reactive protein (CRP) was measured by using an immunonephelometric assay at the Laboratory for Clinical Biochemistry Research at the University of Vermont. C3 and C4 were measured by nephelometry, and double-stranded DNA (dsDNA) antibodies were measured by using the *Crithidia luciliae* method. dsDNA antibodies were considered positive if the titer was at least 1:10.

All patients and controls were examined and validated measures of SLE disease activity (Systemic Lupus Erythematosus Disease Activity Index 2000, or SLEDAI-2K) as well as disease damage (Systemic Lupus International Collaborating Clinics/American College of Rheumatology Damage Index, or SLICC/ACR-DI), which were completed by trained physicians. The SLEDAI-2K and SLICC/ACR-DI instruments were used to calculate disease activity and damage, respectively [23,24]. Disease duration was calculated by using the date the subject fulfilled the 4th ACR classification criteria for SLE for disease onset and the study visit date as the end date. Participants provided information on corticosteroid treatment (current use and duration of treatment) as well as current use of hydroxychloroquine and immunosuppressants (which included cyclophosphamide, azathioprine, methotrexate, mycophenolate mofetil, cyclosporine, and tacrolimus). Renal disease was defined as present if the subject had fulfilled ACR classification criteria for SLE renal involvement (greater than 0.5 gm/day or 3 or more proteinuria or the presence of cellular casts or a combination of these) or had a renal biopsy with evidence of World Health Organization class IIb, III, IV, or V SLE nephritis. International Society of Nephrology/Renal Pathology Society classification was not used, because most renal biopsies were obtained prior to its widespread use.

All 36 individuals underwent imaging evaluations of their carotid arteries, coronary arteries, and aorta. For the purpose of this study, we defined an atherosclerosis phenotype by the presence of at least three of the following four abnormalities on carotid ultrasound or electron beam computed tomography (EBCT): a score of more than 0 on the carotid plaque index, an intima-media thickness (IMT) of greater than mean of the study group (0.67 mm), higher coronary artery calcium (CAC) score (>10), or higher aorta calcium (AC) score (>100) [25,26].

In the coronary arteries and aorta, EBCT scanning was performed to measure vascular calcium by using an Imatron C-150 Ultrafast CT scanner (General Electric Medical Systems, South San Francisco, CA, USA). Calcium scores were calculated with a densitometric program available on the Imatron C-150 scanner by using the Agatston method. The outcome measures used for analyses were dichotomized with a low CAC score of not more than 10 and an AC score of not more than 100 and a high CAC score of greater than 10 and an AC score of greater than 100 signifying the presence of atherosclerosis at these arterial beds [25,26].

Subclinical cardiovascular disease was measured in the carotid arteries by B-mode ultrasound performed by trained sonographers. Carotid ultrasound was performed, and B-mode images of the right and left carotid artery, carotid bifurcation, and the first centimeter of the internal carotid were obtained in multiple planes and assessed for plaque. Carotid ultrasound was completed in a single center. Ultrasonographic measurements were performed by using a Siemens Sequioa model C256 (Siemens, Munich, Germany) equipped with a transducer 8 L5. Sonographers scanned the right and left common carotid artery, carotid bulb, and the first 1.5 cm of the internal and external carotid arteries. For each location, the sonographer imaged the vessel in multiple planes. The sonographers scored the ultrasound images for plaque in the distal common, carotid bulb, and internal arteries. Plaque was defined as a distinct area protruding into the vessel lumen with at least 50% greater thickness than that found in surrounding areas. IMT was measured by using specialized reading software across 1-cm segments of both the right and left sides of the near and far walls of the distal common carotid artery and the far wall of the carotid bulb and internal carotid artery. The mean of all average IMT readings across the eight sites was used for analysis. Reproducibility of IMT and plaque was assessed in five women who underwent two ultrasound examinations within 1 week. Each time, the scanning was performed by a different sonographer, and each scan was scored by two readers. Sonographers and readers were trained as part of a reproducibility protocol study in carotid duplex scanning [27].

Mononuclear cells were isolated from peripheral blood of SLE patients and healthy controls, and then monocytes were isolated and *in vitro*-differentiated into macrophages [20]. Specifically, peripheral blood was drawn in tubes containing citrate-phosphate-dextrose anti-coagulant solution (Sigma-Aldrich, St. Louis, MO, USA). Mononuclear cells were isolated by Histopaque-1077 density gradient centrifugation. Two different approaches were applied to isolate monocytes. The first approach employed a Human CD14 Positive Selection Kit (StemCell Technologies, Vancouver, BC, Canada), which isolates monocytes by directly binding to anti-CD14 antibody-coupled magnetic beads. The cells attached to the beads are immediately frozen in Trizol at -70°C for future RNA isolation. Monocytes obtained from this approach were more than 95% pure. To isolate monocytes for *in vitro* differentiation into macrophages, negative selection was employed (Human Monocyte Enrichment Kit Without CD16 Deletion) and resulted in improved survival during differentiation. The mononuclear cells were incubated with an antibody cocktail including antibodies to CD2, CD3, CD20, CD56, CD66b, CD123, and glycophorin attached to magnetic beads, which were employed because they resulted in higher purity. Cells positive for these markers were depleted, leaving CD14^+^ monocytes from peripheral blood. The isolated CD14^+^ cells were allowed to attach to plastic for 1 hour and then were *in vitro*-differentiated in RPMI-1640 culture medium supplemented with 20% fetal bovine serum, 10 ng/mL macrophage colony-stimulating factor, 1 μg/mL polymyxin-B, 100 U penicillin, and 100 mg/mL streptomycin for 7 days at 37°C in 5% CO_2_.

RNA was extracted from monocytes and macrophages by Trizol reagent (Invitrogen, Carlsbad, CA, USA). The quality of RNA was determined by 2100 Bioanalyzer (Agilent Technologies, Santa Clara, CA, USA), evaluated by the RNA integrity number of at least 8.0 and the ratio of 28 s and 18 s ribosomal RNA. RNA that passed quality control was then used to create cDNA and biotinylated cRNA by using an Illumina TotalPrep RNA Amplification Kit (Illumina, San Diego, CA, USA). RNA was hybridized to a Sentrix^®^ Human-6 Expression BeadChip (Illumina) to measure expression of more than 47,000 transcripts in each sample.

Microarray data were normalized and corrected for batch effect; we then performed significance analysis of microarrays (SAM), multidimensional scaling (MDS) analysis, cluster analysis, and gene ontology (GO) analysis. These analyses were performed first in monocytes and macrophages separately and then during monocyte-to-macrophage differentiation (the difference in expression between an individual’s monocytes and macrophages). For each of these groups, the comparisons included were (a) SLE versus controls, (b) all individuals with versus without an atherosclerosis phenotype, and (c) SLE patients with versus without an atherosclerosis phenotype.

The microarray data were normalized by a quantile normalization procedure by using the bioconductor package affy, and batch effect was corrected by using the ComBat algorithm [28]. Cluster analysis was performed to identify gene expression patterns by using CLUSTER and TREEVIEW software. GO analysis was performed by using the online application PANTHER (Protein Analysis Through Evolutionary Relationships) [29]. The significance of enrichment of differentially expressed genes for each GO term was determined by the Bonferroni-corrected chi-squared *P* value as the default setting in PANTHER, whereas the significance of differential expression was determined by nominal *P* value of less than 0.05 from the *t* test and a fold change of greater than 2. MDS analysis was performed to reveal the sample relation by using global gene expression profiles. For monocyte-to-macrophage differentiation analysis, we calculated fold change for each probe as the ratio of macrophage expression over monocyte expression from each pair of monocyte-macrophage samples (from the same subject). With these fold-change data, we excluded transcripts that have average fold changes of less than 2 in both SLE patient and control groups. This filtering process resulted in 3,044 (out of 47,231) transcripts for data analysis. To determine the fold-change difference between SLE patients and controls for each of these 3,044 transcripts, we calculated the *P* value by using the two-sample *t* test. The microarray data are uploaded to Gene Expression Omnibus with accession number GSE37356.

## Results

### Demographic, clinical, laboratory, and imaging results

Overall, there were few differences between baseline patient and control characteristics. Ten (50%) of 20 patients with SLE and 10 (62.5%) of 16 controls had three of four imaging abnormalities and were considered to have an atherosclerosis phenotype. Patients with SLE were younger than controls: means of 46.9 versus 52.8 years, *P* = 0.09 (not significant). No patients or controls had prior cardiovascular or cerebrovascular events attributable to atherosclerosis (two patients with SLE had prior stroke related to antiphospholipid antibody syndrome). Further clinical, demographic, and imaging data are detailed in Table 1. Patients with SLE had long-standing disease with a mean 16.3-year duration and historically fulfilled a median of five ACR classification criteria, 45% had anti-dsDNA antibodies, and on average patients had some damage (SLICC/ACR-DI mean of 2.15 ± 1.81). The majority of patients (65%) were taking hydroxychloroquine, 30% were on corticosteroids, and 40% were on immunosuppressive agents (Table 1). With these medications, disease at the time of the study visit was largely inactive or mild, and SLEDAI scores averaged 4.2.

**Table 1:** Demographic, clinical, laboratory, medication, and imaging data from 20 systemic lupus erythematosus patients and 16 controls

	**SLE mean ± SD**	**Control mean ± SD**	***p *** **value**
Age, years	46.9 ± 8.9	52.8 ± 11.0	0.09
Race, % who were Caucasian	75	80	0.98
Body mass index, kg/m^2^	27.7 ± 7.7	28.3 ± 4.9	0.79
Waist-hip ratio	0.85 ± 0.07	0.86 ± 0.08	0.69
Systolic blood pressure, mm Hg	117.4 ± 13.2	116.8 ± 14.4	0.88
Diastolic blood pressure, mm Hg	72.3 ± 9.0	71.8 ± 11.5	0.79
Current smoking, %	20	26.7	0.65
Diabetes, %	5	6	0.93
Family history of cardiovascular disease, %	10	7	0.74
Menopausal, %	45	53	0.64
Cardiovascular events	0	0	n/a
Total cholesterol, mg/dL	188.9 ± 51.9	218.9 ± 41.4	0.12
LDL_c_, mg/dL	101.5 ± 41.5	139.0 ± 8.7	0.04
Triglycerides, mg/dL	125.6 ± 99.9	101.0 ± 48.9	0.44
Glucose, mg/dL	98.4 ± 10.7	103.7 ± 13.9	0.27
Glomerular filtration rate, mL/min	80.7 ± 17.8	77.9 ± 18.8	0.65
C-reactive protein, mg/L	3.5 ± 3.6	3.8 ± 6.3	0.91
C3, mg/dL	101.5 ± 21.5		
C4, mg/dL	20.3 ± 8.4		
dsDNA (crithidia) level	66.3		
Presence of carotid plaque	45	53	0.65
Higher CAC score (>10), %	35	33	0.92
Higher AC score (>100), %	50	47	0.85
Intima-media thickness (mean ± SD)	0.65 ± 0.14	0.71 ± 0.21	0.27
Atherosclerosis phenotype, %	50	62.5	0.47
SLEDAI-2 K	4.2 ± 4.3		
SLICC/ACR-DI	2.2 ± 1.8		
Disease duration, years	16.3 ± 8.2		
Total ACR SLE classification criteria (median)	5		
Corticosteroids	30	0	0.01
Hydroxychloroquine	65	0	0.00001
Immunosuppressants	40	0	0.002
Statins	35	0	0.03
Antihypertensives	45	25	0.09

Atherosclerosis phenotype was defined as the presence of at least three of the following four abnormalities on carotid ultrasound or electron beam computed tomography: presence of carotid plaque, intima-media thickness greater than mean of the study group, high coronary calcium score of more than 10, or high aorta calcium score of more than 100. Cardiovascular events were defined as myocardial infarction, coronary artery bypass surgery, coronary intervention, or cerebrovascular events (transient ischemic attack or stroke) related to atherosclerotic disease. Validated measures of lupus disease activity and disease damage (SLEDAI-2 K and SLICC/ACR-DI) were completed by trained physicians. The disease duration was calculated by using the date the subject fulfilled the 4th American College of Rheumatology (ACR) classification criteria for lupus as onset date and study visit date as the end date. Renal disease was defined as being present if the subject had fulfilled ACR classification criteria for lupus renal involvement (greater than 0.5 g/day, 3+ proteinuria, and/or the presence of cellular casts) or had a renal biopsy with evidence of World Health Organization Class IIb, III, IV, or V lupus nephritis. The column on the right denotes percentages of patients meeting the various ACR clinical classification for systemic lupus erythematosus (SLE). Double-stranded DNA (dsDNA) antibody level average includes only patients in whom these antibodies were present. Current smoking was defined as individuals reporting use of one or more cigarettes daily, and no individuals reported prior smoking history. p values were calculated by using a Student’s *7t*-test. AC, aortic calcium; CAC, coronary artery calcium; LDLc, low-density lipoprotein cholesterol; SD, standard deviation; SLEDAI-2 K, Systemic Lupus Erythematosus Disease Activity Index-2000; SLICC/ACR-DI, Systemic Lupus International Collaborating Clinics/American College of Rheumatology Damage Index.

### Gene expression profiles in monocytes and monocyte-derived macrophages

**Figure 1:**
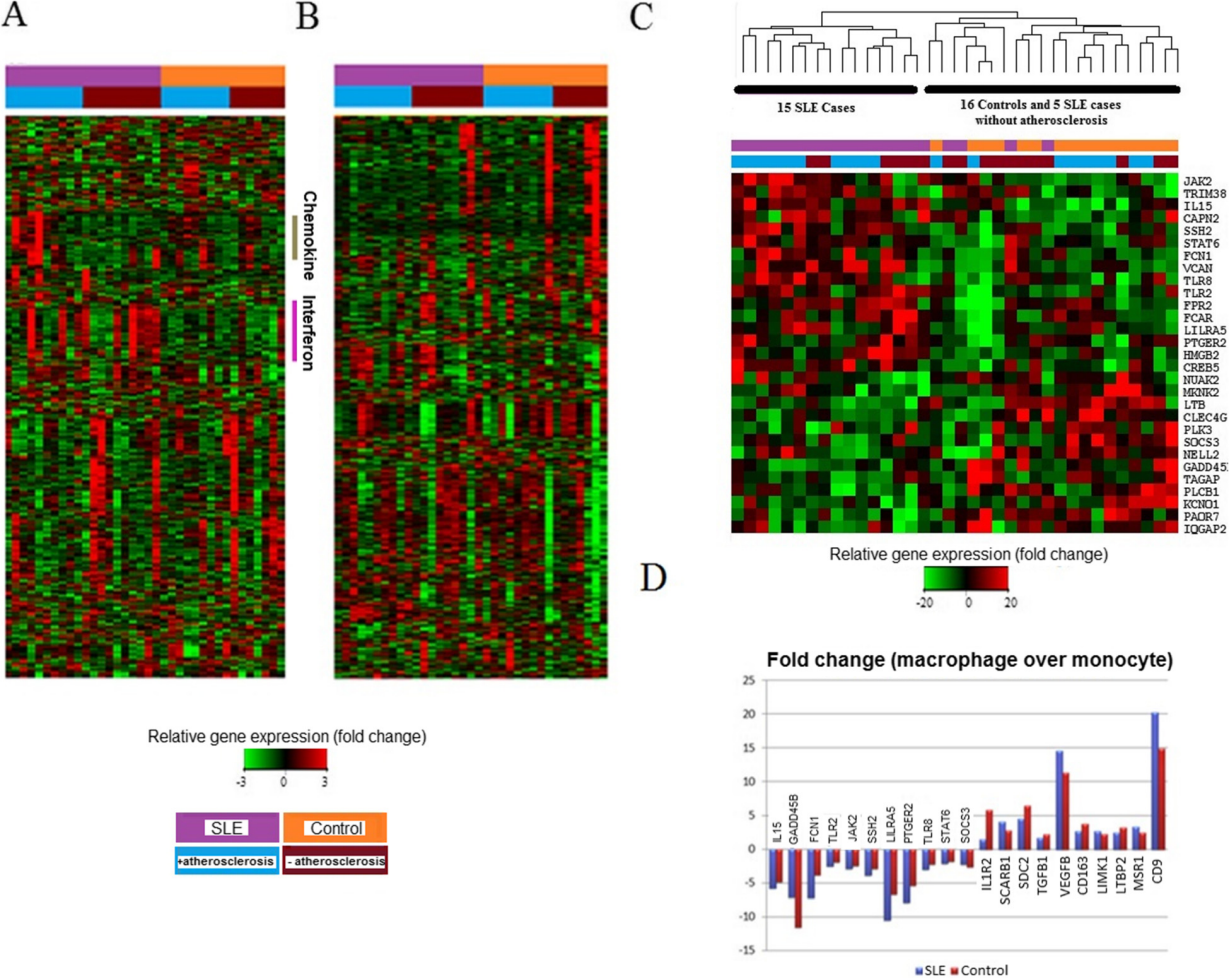
**Gene expression profiles in monocytes, macrophages, and monocyte-macrophage differentiation. (A,B)** Gene expression profiles using cluster analysis of 1,000 genes with highest coefficients of variation arranged by disease status and atherosclerosis phenotype. Panel **(A)** shows expression patterns in monocytes, and panel **(B)** shows expression in macrophages. Above each heatmap is a legend: in the upper section, the purple line denotes patients with systemic lupus erythematosus (SLE) and the orange line denotes healthy controls; in the lower section, the blue line denotes individuals with an atherosclerosis phenotype (as defined in the methods section) and the maroon line denotes individuals without the atherosclerosis phenotype. In the monocyte heatmap, note the presence of the interferon signature (denoted by pink bar to right) in nine out of 20 SLE patients and the chemokine signature (brown bar) in three patients, both of which are very enriched compared with controls. **(C)** Representative heatmap demonstrating downregulated signal transduction genes during monocyte-to-macrophage differentiation after pairwise comparison of monocyte and macrophage expression in all 36 samples. Red (green) pixels indicate more (less) upregulation of expression in macrophages compared with monocytes. **(D)** Histogram showing fold change (both upregulated and downregulated genes) among genes involved in signal transduction pathway during monocyte-to-macrophage differentiation.

The rank-based permutation method SAM did not identify any discernible pattern of differential expression between patients and controls or individuals with and without atherosclerosis in either monocytes or monocyte-derived macrophages (*p* >0.05 for all comparisons). However, striking expression differences were evident between a subgroup of SLE patients and controls from the primary monocyte data (Figure 1A). The most prominent signature seen represents interferon-inducible genes in concordance with previous microarray data from patients with SLE [12,14]. The interferon-inducible gene signature was prominent in nine out of 20 cases and two out of 16 controls (odds ratio 5.7, 95% confidence interval (CI) 1.02, 32.0) (Figure 1A). One case and one control individual with a marginal interferon signature visually were considered not to have the signature, because they had less than 1.5 fold-change upregulation of greater than half of the 53 interferon-inducible genes. Of the nine SLE patients with the interferon signature, three had atherosclerosis; neither of the two controls with the interferon signature had atherosclerosis. This signature was also present in macrophages, although it was less prominent (Additional file 1). Another small subset of patients with SLE (four out of 20 patients) did not have the interferon-inducible signature but instead had upregulation of gene expression levels in a group of genes which encode largely for chemokines (CXCL2, CCL3, CCL14, and CCL20) and other soluble pro-inflammatory molecules: tumor necrosis factor, interleukin-1 (IL-1), and IL-6. One of four individuals with this chemokine signature had atherosclerosis. One individual had both the interferon and chemokine signatures. The remaining patients did not have any gene signature that differentiated them from controls. The full list of genes identified in the interferon and chemokine gene signatures is detailed in Additional files 2 and 3.

### Monocyte-to-macrophage differentiation

When we compared each individual’s macrophage and monocyte gene expression levels, we identified 3,044 genes with a macrophage over monocyte fold change with a magnitude of greater than 2 in either the SLE group or control group. A two-sample *t* test was performed to determine the fold-change difference between SLE patients and controls. In this analysis, 270 transcripts (8.9%) had more fold change during monocyte-to-macrophage differentiation in SLE patients compared with controls than would be expected by chance (*p* <0.05). Employing GO analysis by using the PANTHER database, we found significant (*p* <0.001) enrichment of genes involved in signal transduction, immune system processes, carbohydrate and lipid metabolic processes, and apoptosis differentially regulated between the patients and controls (Table 2 and Additional files 4 and 5).

**Table 2:** Biologic processes in which genes associated with differential expression during monocyte-to-macrophage differentiation

**Biological process**	**Genes in PANTHER database**	**Observed**	**Expected**	***χ***^**2 **^ ***p *** **value**
Immune system process	2,628	127	52.93	9.98 × 10^-22^
Signal transduction	4,191	147	84.41	5.75 × 10^-13^
Apoptosis	966	45	19.45	2.18 × 10^-7^
B cell-mediated immunity	314	21	6.32	2.49 × 10^-6^
Lipid metabolic process	1,119	46	22.54	4.59 × 10^-6^
Intracellular signaling	1,568	58	31.58	6.08 × 10^-6^
Macrophage activation	305	19	6.14	1.97 × 10^-5^
Response to interferon-gamma	105	10	2.11	6.83 × 10^-5^
Induction of apoptosis	358	19	7.21	1.58 × 10^-4^
Carbohydrate metabolism	952	35	19.17	5.33 × 10^-4^
Complement activation	162	11	3.26	5.43 × 10^-4^

PANTHER, Protein Analysis Through Evolutionary Relationships.

Of the biologically relevant pathways observed, the signal transduction pathway contained 147 differentially regulated genes, the most of any pathway (Table 2). We looked at the expression pattern of differentially expressed genes in this pathway and found that there were clear differences between the SLE patients and the controls for genes that were either upregulated or downregulated during macrophage differentiation (Figure 1C, D). When cluster analysis was performed with both upregulated and downregulated genes in this pathway, SLE patients and controls were distinguishable. In the case of downregulated genes, the clusters were able to differentiate 15 of the 20 patients from all of the 16 controls and five SLE patients of whom none had an atherosclerosis phenotype (Figure 1C). This pattern was not seen when genes from other relevant pathways were analyzed.

### Correlations with atherosclerosis

Unsupervised expression profiling in monocytes and macrophages by using the 1,000 most differentially expressed genes—those with the highest coefficient of variation (C_v_)—was unable to clearly distinguish individuals with and without atherosclerosis. However, in monocytes from patients with SLE, we demonstrated that patients with subclinical atherosclerosis demonstrated substantial enrichment of genes identified as associated with atherosclerosis based on a previously described 344-gene signature used to differentiate individuals with and without a substantial burden of atherosclerosis [18]. A complete gene list for this signature is detailed in Additional file 6. When used to look at all individuals, this signature did not effectively delineate disease status or atherosclerosis status (Additional file 7). We found that only 140 (4.6%) of 3,044 genes demonstrated a *P* value of less than 0.05 when comparing the 19 participants with atherosclerosis with the 17 participants without atherosclerosis (Additional file 3A). Among the 20 patients with SLE, we found 163 significant genes (5.3%) with a *P* value of less than 0.05 between those with or without atherosclerosis (Additional file 3B). Among the 16 controls, we found only 74 (2.4%) genes with a *P* value of less than 0.05 between those with or without atherosclerosis (Additional file 3C). Of the genes identified in each of these analyses, 11 (7.8%) out of 140, 22 (13.5%) out of 163 genes, and three (1.3%) out of 74 overlapped the previously identified 344-gene atherosclerosis signature (Additional file 3D). Upon hierarchical clustering analysis, these differentially expressed genes did not differentiate any of the groups by atherosclerosis status.

**Figure 2:**
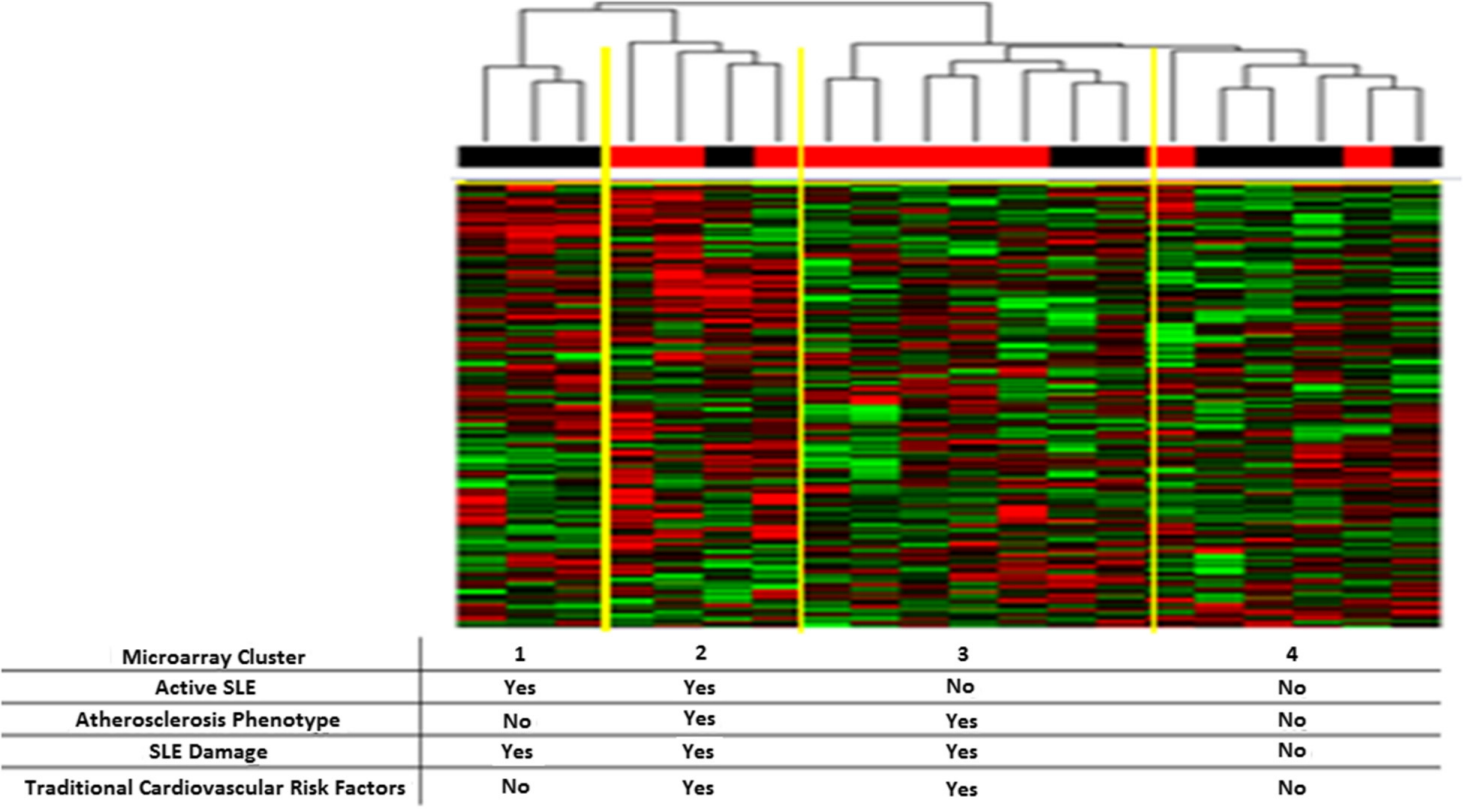
**Tree dendrogram derived from expression patterns of a previously described 344-gene atherosclerosis signature in systemic lupus erythematosus (SLE) patients.** Red boxes above an individual’s expression heatmap represent the presence of the atherosclerosis phenotype, and a black box denotes its absence. Below each cluster, the individuals are stratified as having or not having active SLE (average SLEDAI of more than 4), atherosclerosis phenotype (groups identified with the phenotype had greater than 70% of individuals with the atherosclerosis phenotype), SLE damage (average SLICC/ACR-DI of more than 2), and traditional cardiovascular risk factors, including an average of at least one of the four risk factors of hypertension, dyslipidemia, diabetes, and smoking. SLEDAI, Systemic Lupus Erythematosus Disease Activity Index; SLICC/ACR-DI, Systemic Lupus International Collaborating Clinics/American College of Rheumatology Damage Index.

We found that the 344-gene atherosclerosis signature hierarchically clustered patients with SLE into four clusters based on disease activity and atherosclerosis status (Figure 2 and Table 3). Cluster 1 had active SLE (defined as SLEDAI ≥4) with minimal atherosclerosis and cardiovascular risk factors, cluster 2 had both active SLE and atherosclerosis with more traditional cardiovascular risk factors, cluster 3 had inactive SLE but substantial atherosclerosis and traditional risk factors, and cluster 4 had neither active SLE nor atherosclerosis but minimal cardiovascular risk factors. Although the clustering first separated individuals by disease activity (clusters 1 and 2 SLEDAI = 7.7 ± 4.7, clusters 3 and 4 SLEDAI = 2.3 ± 2.7), within these clusters, clusters 2 and 3 were highly enriched for atherosclerosis (eight of 11 patients had the atherosclerosis phenotype) whereas clusters 1 and 4 were composed primarily of those without the atherosclerosis phenotype (two of nine patients had the atherosclerosis phenotype). When comparing the individuals with atherosclerosis (clusters 2 and 3) with the individuals without atherosclerosis (clusters 1 and 4), we found an odds ratio of 9.3 (95% CI 1.2, 72.9) for this signature. We also confirmed our findings by using a resampling-based consensus clustering analysis with these genes (Additional file 8).

**Table 3:** Clinical characteristics of patients stratified by microarray clusters using 344-gene atherosclerosis signature

	**Microarray cluster**	**1**	**2**	**3**	**4**
Demographics	Age	45.8 ± 9.3	46.9 ± 5.4	49.0 ± 7.4	45.0 ± 13.0
	Disease duration	13.6 ± 9.6	21.2 ± 3.31	17.3 ± 8.8	13.1 ± 9.2
SLE disease activity/damage	SLEDAI-2 K	8.7 ± 6.4	7.0 ± 3.8	2.0 ± 3.1	2.7 ± 2.4
	SLICC/ACR-DI	2.3 ± 2.3	4.0 ± 0.8	2.3 ± 1.6	0.7 ± 1.2
	Renal disease	67.6%	100%	14.3%	33.3%
Atherosclerosis	Atherosclerosis phenotype	0%	75.0%	71.0%	20.0%
	Carotid intima-media thickness	0.53	0.69	0.72	0.59
	Carotid plaque	0%	75.0%	57.1%	33.3%
	Coronary artery calcium score	0	124 ± 85	349 ± 786	103 ± 252
	Aortic calcium score	0	2,638 ± 3,261	828 ± 995	89 ± 149
Traditional cardiovascular risk factors	0	33.3%	25%	28.6%	66.7%
	1	66.7%	25%	14.3%	16.7%
	2	0%	25%	28.6%	16.7%
	3	0%	25%	28.6%	0%
	Average number of risk factors	0.7	1.5	1.6	0.5
Current medication use	Steroids	33.3%	75.0%	28.6%	0.0%
	Hydroxychloroquine	100%	75.0%	71.4%	33.3%
	Immunosuppressants	33.3%	100%	28.6%	16.7%
	Statins	0%	75.0%	28.6%	33.3%

Demographics, disease activity, and atherosclerosis measures are given in aggregate for each cluster. The four traditional cardiovascular risk factors assessed were hypertension (defined as either systolic blood pressure of more than 140 mm Hg, diastolic blood pressure of more than 90 mm Hg or use of antihypertensives and excluded individuals taking antihypertensives only for renal protective measures in the setting of systemic lupus erythematosus (SLE) nephritis), dyslipidemia (defined as elevated total cholesterol or low-density lipoprotein according to Adult Treatment Panel III guidelines [30] or use of cholesterol lowering medication), diabetes, and smoking. Definition and assessment of imaging parameters are described in the Materials and methods. SLEDAI-2 K, Systemic Lupus Erythematosus Disease Activity Index-2000; SLICC/ACR-DI, Systemic Lupus International Collaborating Clinics/American College of Rheumatology Damage Index.

## Discussion

When we compared global gene expression of patients with SLE and controls, we confirmed the importance of type-1 interferon signature in monocytes. In addition, we found that signal transduction, macrophage activation, response to interferon-gamma, and apoptosis were differentially regulated during the process of monocyte-to-macrophage differentiation.

About half of patients possess the interferon signature and it appears to be associated with more severe disease, including renal disease, which is a leading cause of morbidity and mortality in SLE. Although previous work has shown a mechanistic link between interferon-alpha and atherosclerosis in SLE with interferon promoting macrophage lipid uptake and foam cell formation [6], interestingly, we did not see an association between this signature and atherosclerosis and instead saw that atherosclerosis was more common in individuals who did not have an interferon signature with seven out of 11 without the interferon signature having atherosclerosis versus two out of nine among individuals with the interferon signature. Although the number of patients who expressed a cytokine/chemokine expression signature is small (four out of 20), the genes seen differentially expressed in this subgroup suggest that alteration in innate rather than adaptive immunity may play an important role in the pathogenesis of SLE in a subset of patients. In addition to having high disease activity, these individuals tended to have slightly shorter disease duration, and none had an atherosclerosis phenotype (Table 3). Although larger studies will be needed to confirm these types of gene expression signature associations with clinical data, if robust, they could potentially be used help stratify patients’ risk for various disease manifestations or risk of atherosclerosis.

Interestingly, we also identified lipid and carbohydrate metabolism genes as differentially expressed in SLE patients compared with controls. One could hypothesize that these genes’ expression may play a role in how these cells participate in the processes of energy metabolism and mitochondrial function during cellular differentiation or during atheroma formation. These observations suggest that monocytes from patients with SLE upon entering sites of inflammation or vascular injury may be programmed as they differentiate into macrophages with an enhanced pathogenic potential to promote an atherosclerosis phenotype.

Signal transduction genes during monocyte-to-macrophage differentiation were predictive of the presence or absence of SLE; differentially downregulated genes were able to identify 15 of 20 SLE patients from the controls and five SLE patients who did not have atherosclerosis (Figure 1B). Many of the genes differentially regulated during monocyte-to-macrophage differentiation (downregulated genes include *JAK2*, *STAT6*, *TLR8*, and *TLR*2, and upregulated genes include *VEGFB*, *TGFB1*, *FN1*, *IL-1R2*, *SCARB1*, *MSR1*, and *CD163*) have been previously reported in the pathogenesis of atherosclerosis [16,18,31,32].

Foamy macrophages in atherosclerotic plaques demonstrate both M1 and M2 markers, and similar trends were observed during monocyte-to-macrophage differentiation in SLE. Mechanistically, we found that *JAK2* and *STAT6* were suppressed to a greater degree during monocyte-to-macrophage differentiation in patients with SLE compared with controls, especially among patients with atherosclerosis. *JAK2* and *STAT6* are both important in mediating M2 macrophage differentiation [33]. Expression of *SOCS3*, a JAK/STAT suppressor whose deficiency leads to M1 differentiation, was also reduced to a greater degree in patients with SLE. Furthermore, *IL15*, which mediates its signal through STATs including *STAT6*, was reduced more during monocyte-to-macrophage differentiation in patients with SLE, especially in those with atherosclerosis. The JAK2 fold-change expression was highly correlated with *IL15* and *IFITM1*, which supports the internal consistency of the data. These results indicate that SLE patients’ monocytes favor M1 rather than M2 differentiation compared with controls. Furthermore, we found that, compared with controls, patients with SLE had significantly increased levels of vascular endothelial growth factor (*VEGFB*) which promotes ischemic myocardial revascularization [31]. CD163, an M2 marker, is highly expressed in atherosclerotic plaques, whereas MSR1 is important in plaque formation [32,34]. Patients with SLE therefore appear to have an increased predilection for creating inflammatory M1 macrophages but also exhibit features associated with M2 differentiation which may contribute to the pathogenesis of atherosclerosis.

One inherent limitation of the present study is the small sample size. Although clinical heterogeneity in SLE is also a concern, the SLE patients analyzed in this study were representative of the larger SOLVABLE population of 165 individuals who did not participate in this study in terms of clinical parameters and medication use. Because of potential confounding from the relatively small sample size, we looked at other variables, including medication usage, given the higher use of immunomodulatory medications and statin medications in the patients with SLE. Although these differences may contribute to some of the differences observed, we did not find that individuals’ expression clustered by medication use, and clustering based on medication status was not able to identify patients with or without atherosclerosis (data not shown). Given that patients on medication still had more severe disease, the medications may have dampened these patients’ inflammatory signatures.

Monocyte gene expression patterns of patients with SLE and subclinical atherosclerosis were associated with a 344-gene signature previously reported as associated with atherosclerosis (Figure 2) [18]. This signature includes primarily immune and inflammatory genes, including six Toll-like receptor genes, *IL1B*, *IRAK3*, and *MAPK14*, and a number of genes involved in apoptosis and mobilization of calcium. Strikingly, hierarchical clustering using this gene signature divided patients into groups based on their disease activity, and within these clusters, those with and without an atherosclerosis phenotype. Although the presence/absence of atherosclerosis was not absolute in any group, the differences between groups were readily apparent. Differences in patient demographics such as age and disease duration were not statistically significant and cannot fully account for the differences we saw between clusters in terms of atherosclerosis. We did note a trend (*p* = 0.14) for clusters 2 and 3 to have longer standing disease, and therefore without longitudinal follow-up of clusters 1 and 4, it is difficult to say whether these individuals will develop atherosclerosis at similar rates once they have had longer disease duration. Cluster 2 (high disease activity, 100% renal disease, and abundant atherosclerosis), like cluster 3 (low disease activity and abundant atherosclerosis), had substantial traditional cardiovascular risk factors (diabetes, smoking, dyslipidemia, and hypertension). The data suggest that both disease activity and traditional risk factors appear to contribute to cardiovascular risk in patients with SLE and raise the question of whether these risks are additive. One potential confounder for this gene expression pattern could be medication use, especially because immune modulating medications may affect the expression of genes relevant to the signatures being examined. For example, patients in cluster 2 were most likely to be on steroids (*P* = 0.03) and immunosuppressants (*p* = 0.004), which could affect their expression profile. Also, the use of statins in patients with SLE may have had an effect on gene expression and was likely to have had an effect on progression (and possibly development) of atherosclerosis [35]. These data are intriguing and hypothesis-generating but will need to be validated in a larger cohort of patients than was possible in this pilot study.

## Conclusions

Our data support the importance of the interferon-inducible signature in SLE and suggest that gene expression profiles in monocytes from patients with SLE are different between individuals with high and low disease activity and with and without atherosclerosis. The changes in expression seen during monocyte-to-macrophage differentiation suggest that this process of differentiation may contribute to disease pathogenesis, possibly by polarizing macrophages toward classic M1 activation. Further studies may be directed to determine whether factors present in SLE serum, genetic predisposition, or stimuli such as the stress response of oxidized lipid uptake might more clearly identify factors that promote the differentiation of pathogenic macrophages in SLE.

## Abbreviations

AC: aorta calcium; ACR: American College of Rheumatology; CAC: coronary artery calcium; CI: confidence interval; CT: computerized tomography; dsDNA: double-stranded DNA; EBCT: electron beam computed tomography; GO: gene ontology; IL: interleukin; IMT: intima-media thickness (carotid); LDL-c: low-density lipoprotein cholesterol; PANTHER: Protein Analysis Through Evolutionary Relationships; SAM: significance analysis of microarrays; SLE: systemic lupus erythematosus; SLEDAI: systemic lupus erythematosus disease activity index; SLICC/ACR-DI: Systemic Lupus International Collaborating Clinics/American College of Rheumatology Damage Index; SOLVABLE: Study of Lupus Vascular and Bone Long-term Endpoints; *VEGFB*: vascular endothelial growth factor.

## Competing interests

The authors declare that they have no competing interests.

## Authors’ contributions

BK participated in study design and coordination, performed analysis of the expression data and clinical data, and drafted the manuscript. C-CH performed statistical analysis of expression data and participated in study coordination. CS and PW participated in study design and coordination and performed clinical data collection. RK, DY, and QQH carried out the *in vitro* studies and RNA preparation. KS-T, WP, GK, and DE performed and interpreted the clinical imaging studies. RP and RR-G conceived of the study, participated in its design and coordination and in interpretation of the data, and helped to draft the manuscript. All authors read and approved the final manuscript.

## Supplementary Material

Additional file 1 **Macrophage Interferon Signature.** Heatmap of limited interferon signature identified in *in vitro*-differentiated macrophages is shown. SLE, systemic lupus erythematosus. Click here for file

Additional file 2 **Interferon gene signature.** Expression values for each of the 53 interferon-inducible genes identified are shown. Click here for file

Additional file 3 **Monocyte-to-macrophage differentiation.** Genes differentially expressed during monocyte-to-macrophage differentiation are shown. Click here for file

Additional file 4 **Gene networks.** Significant gene interaction networks were determined by using pathway analysis performed by using Ingenuity Pathways Analysis (IPA). GO, gene ontology; PANTHER, Protein Analysis Through Evolutionary Relationships. Click here for file

Additional file 5 **Gene ontology (GO) term breakdowns.** Heatmaps derived from genes representing significant GO terms for all systemic lupus erythematosus (SLE) cases and controls are shown. Click here for file

Additional file 6 **Three hundred forty-four-gene atherosclerosis signature.** Gene list for previously defined 344-gene atherosclerosis gene signature is shown. Click here for file

Additional file 7 **Three hundred forty-four-gene atherosclerosis signature.** Heatmap demonstrates previously described 344-atherosclerosis gene signature in all systemic lupus erythematosus (SLE) cases and controls. Click here for file

Additional file 8 **Consensus clustering.** Resampling-based consensus clustering analysis for 344-gene atherosclerosis signature is shown. Click here for file
