# Supplementary material for: Inflammatory expression profiles in monocyte-to-macrophage differentiation in patients with systemic lupus erythematosus and relationship with atherosclerosis
Source: Arthritis Res Ther. 2014 Jul 10;16(4):R147. doi: 10.1186/ar4609 (PMC4227297; doi:10.1186/ar4609)

**Supplemental figure 1.** Limited interferon signature identified in in-vitro differentiated macrophages. SLE patients are denoted with a ‘C’ while healthy controls are denoted with an ‘X’. Presence of the atherosclerosis phenotype is denoted with an ‘A’ while its absence is denoted with an ‘N’.


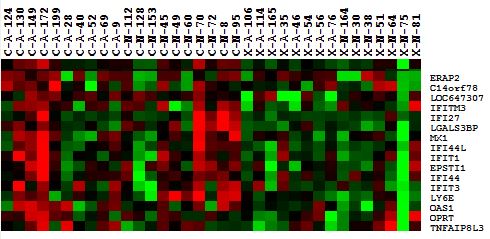

Supplement: Additional file 1 — Macrophage Interferon Signature. Heatmap of limited interferon signature identified in in vitro-differentiated macrophages is shown. SLE, systemic lupus erythematosus. [file ar4609-S1.docx]
