# Supplementary material for: Inflammatory expression profiles in monocyte-to-macrophage differentiation in patients with systemic lupus erythematosus and relationship with atherosclerosis
Source: Arthritis Res Ther. 2014 Jul 10;16(4):R147. doi: 10.1186/ar4609 (PMC4227297; doi:10.1186/ar4609)
Supplement: Additional file 2 — Interferon gene signature. Expression values for each of the 53 interferon-inducible genes identified are shown. [file ar4609-S2.docx]

**Supplementary Table 1.**  Interferon gene signature.  In the right half of the table average log(expression) values are listed for each group for each of the 53 interferon-inducible genes identified. SLE – systemic lupus erythematosus.  Fold change comparisons in the right half of the table indicate the degree to which the gene is differentially regulated between different groups.  Ctrl – control individuals.  SLE-IFN – lupus patients with the interferon signature based on heatmaps.  SLE-No-IFN – lupus patients without the interferon signature.  SLE-A – lupus patients with the atherosclerosis phenotype. SLE-NA – lupus patients without the atherosclerosis phenotype.  Ctrl-A – control individuals with atherosclerosis phenotype.  Ctrl-NA – control individuals without the atherosclerosis phenotype.

|  |  |  | | | | | | | |  |  | | | | | | |
| --- | --- | --- | --- | --- | --- | --- | --- | --- | --- | --- | --- | --- | --- | --- | --- | --- | --- |
|  |  | Expression by Group (Log_2_) | | | | | | | |  | Fold Change Comparisons | | | | | | |
| Gene |  | SLE | Ctrl | SLE-IFN | SLE-No-IFN | SLE-A | SLE-NA | Ctrl-A | Ctrl-NA |  | SLE/Ctrl | SLE-IFN/Ctrl | SLE-IFN/SLE-Non-IFN | SLE-A/SLE-NA | Ctrl-A/Ctrl-NA | SLE-A/Ctrl-A | SLE-NA/Ctrl-NA |
|  |  |  |  |  |  |  |  |  |  |  |  |  |  |  |  |  |  |
| CMPK2 |  | 5.34 | 5.05 | 5.76 | 4.99 | 5.25 | 5.43 | 5.04 | 5.06 |  | 1.22 | 1.64 | 1.71 | 0.88 | 0.98 | 1.15 | 1.29 |
| DDX60 |  | 6.06 | 5.70 | 6.44 | 5.76 | 6.01 | 6.12 | 5.64 | 5.78 |  | 1.29 | 1.67 | 1.60 | 0.93 | 0.91 | 1.29 | 1.27 |
| DHX58 |  | 6.10 | 5.90 | 6.38 | 5.88 | 6.11 | 6.10 | 5.88 | 5.92 |  | 1.15 | 1.40 | 1.42 | 1.01 | 0.97 | 1.17 | 1.13 |
| EIF2AK2 |  | 7.70 | 7.37 | 8.05 | 7.42 | 7.59 | 7.82 | 7.35 | 7.40 |  | 1.26 | 1.60 | 1.55 | 0.85 | 0.97 | 1.18 | 1.34 |
| EPSTI1 |  | 7.99 | 7.51 | 8.50 | 7.56 | 7.97 | 8.00 | 7.52 | 7.49 |  | 1.39 | 1.99 | 1.91 | 0.98 | 1.02 | 1.36 | 1.43 |
| GMPR |  | 5.40 | 5.18 | 5.68 | 5.17 | 5.36 | 5.44 | 5.14 | 5.22 |  | 1.17 | 1.42 | 1.42 | 0.94 | 0.95 | 1.16 | 1.16 |
| HERC5 |  | 7.59 | 7.20 | 8.16 | 7.12 | 7.50 | 7.67 | 7.17 | 7.23 |  | 1.31 | 1.95 | 2.06 | 0.89 | 0.96 | 1.26 | 1.35 |
| HERC6 |  | 6.09 | 5.79 | 6.43 | 5.81 | 6.00 | 6.18 | 5.76 | 5.82 |  | 1.23 | 1.56 | 1.53 | 0.89 | 0.96 | 1.18 | 1.28 |
| HESX1 |  | 5.01 | 4.76 | 5.29 | 4.78 | 4.93 | 5.09 | 4.76 | 4.75 |  | 1.19 | 1.45 | 1.43 | 0.90 | 1.01 | 1.12 | 1.27 |
| HSH2D |  | 6.20 | 5.96 | 6.41 | 6.03 | 6.07 | 6.32 | 5.93 | 6.00 |  | 1.18 | 1.36 | 1.30 | 0.84 | 0.96 | 1.10 | 1.25 |
| IFI27 |  | 5.92 | 4.93 | 6.89 | 5.13 | 5.62 | 6.22 | 4.90 | 4.96 |  | 1.99 | 3.89 | 3.39 | 0.66 | 0.96 | 1.64 | 2.39 |
| IFI44 |  | 8.03 | 7.57 | 8.56 | 7.59 | 8.01 | 8.05 | 7.54 | 7.61 |  | 1.38 | 1.99 | 1.96 | 0.97 | 0.95 | 1.39 | 1.36 |
| IFI44L |  | 7.73 | 7.06 | 8.55 | 7.05 | 7.61 | 7.85 | 7.03 | 7.10 |  | 1.58 | 2.81 | 2.83 | 0.85 | 0.95 | 1.49 | 1.67 |
| IFI6 |  | 7.98 | 7.51 | 8.62 | 7.45 | 7.86 | 8.09 | 7.52 | 7.49 |  | 1.38 | 2.17 | 2.26 | 0.86 | 1.02 | 1.27 | 1.51 |
| IFI6 |  | 8.66 | 8.36 | 9.11 | 8.28 | 8.56 | 8.76 | 8.33 | 8.41 |  | 1.22 | 1.68 | 1.78 | 0.87 | 0.95 | 1.17 | 1.27 |
| IFIH1 |  | 6.46 | 6.21 | 6.79 | 6.19 | 6.40 | 6.51 | 6.24 | 6.17 |  | 1.19 | 1.50 | 1.52 | 0.93 | 1.05 | 1.12 | 1.27 |
| IFIT1 |  | 6.86 | 6.35 | 7.65 | 6.21 | 6.73 | 6.98 | 6.42 | 6.27 |  | 1.42 | 2.47 | 2.73 | 0.84 | 1.11 | 1.24 | 1.65 |
| IFIT2 |  | 7.24 | 6.85 | 7.91 | 6.69 | 7.16 | 7.32 | 6.86 | 6.83 |  | 1.31 | 2.08 | 2.32 | 0.90 | 1.02 | 1.23 | 1.40 |
| IFIT3 |  | 6.21 | 5.67 | 6.87 | 5.67 | 6.07 | 6.35 | 5.70 | 5.62 |  | 1.46 | 2.31 | 2.31 | 0.82 | 1.05 | 1.29 | 1.66 |
| IFITM1 |  | 7.31 | 6.75 | 7.98 | 6.76 | 7.15 | 7.47 | 6.74 | 6.77 |  | 1.47 | 2.34 | 2.33 | 0.80 | 0.97 | 1.33 | 1.62 |
| IFITM3 |  | 8.89 | 8.44 | 9.36 | 8.51 | 8.79 | 8.99 | 8.31 | 8.60 |  | 1.37 | 1.90 | 1.80 | 0.87 | 0.82 | 1.40 | 1.31 |
| IRF7 |  | 7.94 | 7.62 | 8.28 | 7.66 | 7.84 | 8.05 | 7.60 | 7.65 |  | 1.25 | 1.58 | 1.54 | 0.86 | 0.97 | 1.18 | 1.31 |
| ISG15 |  | 7.77 | 7.36 | 8.37 | 7.28 | 7.66 | 7.88 | 7.41 | 7.30 |  | 1.33 | 2.01 | 2.12 | 0.86 | 1.08 | 1.19 | 1.50 |
| LGALS3BP |  | 5.54 | 5.10 | 6.08 | 5.10 | 5.42 | 5.67 | 5.12 | 5.06 |  | 1.36 | 1.98 | 1.97 | 0.84 | 1.04 | 1.23 | 1.52 |
| LY6E |  | 8.15 | 7.84 | 8.69 | 7.71 | 8.08 | 8.22 | 7.82 | 7.87 |  | 1.24 | 1.79 | 1.97 | 0.90 | 0.96 | 1.19 | 1.27 |
| MX1 |  | 8.82 | 8.52 | 9.30 | 8.43 | 8.82 | 8.83 | 8.52 | 8.52 |  | 1.23 | 1.72 | 1.83 | 1.00 | 1.01 | 1.23 | 1.24 |
| MX2 |  | 7.64 | 7.25 | 7.96 | 7.38 | 7.57 | 7.70 | 7.24 | 7.26 |  | 1.31 | 1.64 | 1.49 | 0.91 | 0.99 | 1.26 | 1.36 |
| NEXN |  | 5.76 | 5.56 | 6.09 | 5.49 | 5.67 | 5.86 | 5.59 | 5.51 |  | 1.15 | 1.45 | 1.52 | 0.88 | 1.06 | 1.06 | 1.27 |
| OAS1 |  | 6.66 | 6.30 | 6.96 | 6.42 | 6.69 | 6.63 | 6.36 | 6.21 |  | 1.29 | 1.58 | 1.45 | 1.05 | 1.11 | 1.26 | 1.33 |
| OAS2 |  | 6.12 | 5.76 | 6.58 | 5.74 | 6.06 | 6.18 | 5.74 | 5.78 |  | 1.28 | 1.76 | 1.78 | 0.92 | 0.97 | 1.25 | 1.32 |
| OAS3 |  | 7.37 | 6.98 | 7.88 | 6.95 | 7.26 | 7.47 | 6.99 | 6.96 |  | 1.31 | 1.87 | 1.91 | 0.86 | 1.02 | 1.21 | 1.42 |
| OASL |  | 6.42 | 6.01 | 6.82 | 6.10 | 6.34 | 6.51 | 5.94 | 6.08 |  | 1.34 | 1.75 | 1.64 | 0.89 | 0.91 | 1.31 | 1.34 |
| OTOF |  | 5.60 | 5.07 | 6.29 | 5.03 | 5.41 | 5.79 | 5.06 | 5.08 |  | 1.45 | 2.34 | 2.40 | 0.77 | 0.98 | 1.28 | 1.63 |
| PARP12 |  | 6.98 | 6.74 | 7.32 | 6.70 | 6.95 | 7.00 | 6.72 | 6.77 |  | 1.18 | 1.49 | 1.53 | 0.97 | 0.96 | 1.18 | 1.17 |
| PARP14 |  | 7.06 | 6.87 | 7.38 | 6.80 | 7.02 | 7.10 | 6.84 | 6.90 |  | 1.14 | 1.42 | 1.49 | 0.95 | 0.96 | 1.13 | 1.15 |
| PARP9 |  | 7.16 | 6.79 | 7.62 | 6.78 | 7.17 | 7.14 | 6.80 | 6.77 |  | 1.29 | 1.78 | 1.79 | 1.02 | 1.02 | 1.29 | 1.29 |
| PRIC285 |  | 7.66 | 7.30 | 7.99 | 7.40 | 7.60 | 7.73 | 7.31 | 7.29 |  | 1.28 | 1.61 | 1.50 | 0.91 | 1.02 | 1.22 | 1.36 |
| RABGAP1L |  | 6.68 | 6.53 | 6.92 | 6.48 | 6.65 | 6.71 | 6.51 | 6.55 |  | 1.11 | 1.32 | 1.36 | 0.96 | 0.97 | 1.10 | 1.12 |
| RSAD2 |  | 6.17 | 5.66 | 6.80 | 5.66 | 6.09 | 6.25 | 5.63 | 5.70 |  | 1.42 | 2.20 | 2.21 | 0.90 | 0.96 | 1.37 | 1.46 |
| SAMD9 |  | 6.81 | 6.45 | 7.22 | 6.48 | 6.75 | 6.88 | 6.48 | 6.39 |  | 1.29 | 1.72 | 1.68 | 0.91 | 1.06 | 1.20 | 1.40 |
| SAMD9L |  | 6.82 | 6.36 | 7.31 | 6.42 | 6.83 | 6.81 | 6.37 | 6.35 |  | 1.38 | 1.93 | 1.85 | 1.02 | 1.01 | 1.38 | 1.37 |
| SERPING1 |  | 5.64 | 5.40 | 6.03 | 5.31 | 5.53 | 5.75 | 5.42 | 5.37 |  | 1.18 | 1.55 | 1.65 | 0.85 | 1.03 | 1.08 | 1.30 |
| SERPING1 |  | 4.75 | 4.76 | 4.74 | 4.77 | 4.74 | 4.77 | 4.73 | 4.80 |  | 1.00 | 0.99 | 0.98 | 0.98 | 0.95 | 1.01 | 0.98 |
| SIGLEC1 |  | 5.29 | 4.85 | 5.78 | 4.89 | 5.15 | 5.43 | 4.82 | 4.90 |  | 1.35 | 1.90 | 1.86 | 0.82 | 0.95 | 1.26 | 1.45 |
| SP110 |  | 6.10 | 5.86 | 6.35 | 5.90 | 6.04 | 6.16 | 5.87 | 5.85 |  | 1.18 | 1.40 | 1.37 | 0.92 | 1.01 | 1.12 | 1.24 |
| SPATS2L |  | 5.93 | 5.53 | 6.28 | 5.64 | 5.88 | 5.98 | 5.51 | 5.55 |  | 1.32 | 1.69 | 1.56 | 0.93 | 0.97 | 1.29 | 1.34 |
| TRIM22 |  | 7.51 | 7.16 | 7.80 | 7.27 | 7.52 | 7.50 | 7.14 | 7.20 |  | 1.27 | 1.55 | 1.44 | 1.01 | 0.96 | 1.30 | 1.23 |
| TRIM5 |  | 6.45 | 6.16 | 6.75 | 6.21 | 6.44 | 6.46 | 6.15 | 6.17 |  | 1.22 | 1.50 | 1.45 | 0.98 | 0.99 | 1.22 | 1.22 |
| USP18 |  | 5.23 | 4.87 | 5.70 | 4.84 | 5.07 | 5.39 | 4.86 | 4.89 |  | 1.28 | 1.78 | 1.81 | 0.80 | 0.98 | 1.16 | 1.41 |
| XAF1 |  | 6.75 | 6.52 | 7.10 | 6.47 | 6.66 | 6.84 | 6.57 | 6.44 |  | 1.18 | 1.50 | 1.55 | 0.88 | 1.09 | 1.06 | 1.32 |
| ZBP1 |  | 5.92 | 5.63 | 6.32 | 5.59 | 5.86 | 5.99 | 5.61 | 5.66 |  | 1.22 | 1.62 | 1.66 | 0.91 | 0.97 | 1.19 | 1.26 |
