# Supplementary material for: Inflammatory expression profiles in monocyte-to-macrophage differentiation in patients with systemic lupus erythematosus and relationship with atherosclerosis
Source: Arthritis Res Ther. 2014 Jul 10;16(4):R147. doi: 10.1186/ar4609 (PMC4227297; doi:10.1186/ar4609)
Supplement: Additional file 3 — Monocyte-to-macrophage differentiation. Genes differentially expressed during monocyte-to-macrophage differentiation are shown. [file ar4609-S3.docx]

**Supplemental Table 2.** Genes differentially expressed during monocyte to macrophage differentiation.

A.    140 genes with p<0.05 when comparing macrophage/monocyte expression between the 19 participants with atherosclerosis (A) with the 17 participants without atherosclerosis (NA).

| Gene | A fold change | NA fold change | p-value A vs NA |
| --- | --- | --- | --- |
| FABP4 | 34.012 | 16.420 | 0.003 |
| LOC100130511 | 2.318 | 1.947 | 0.003 |
| ADHFE1 | 0.394 | 0.496 | 0.004 |
| ACAP1 | 0.396 | 0.501 | 0.004 |
| LOC100133866 | 3.953 | 5.744 | 0.005 |
| COTL1 | 0.375 | 0.474 | 0.005 |
| GABBR1 | 0.243 | 0.295 | 0.005 |
| SMAD3 | 0.236 | 0.189 | 0.005 |
| SLC24A4 | 0.293 | 0.237 | 0.005 |
| TUBB1 | 0.302 | 0.559 | 0.005 |
| SLC24A4 | 0.317 | 0.268 | 0.006 |
| LOC347376 | 0.341 | 0.411 | 0.006 |
| LOC728457 | 0.422 | 0.558 | 0.006 |
| CAMKK2 | 0.370 | 0.464 | 0.006 |
| LOC652616 | 0.190 | 0.290 | 0.007 |
| IL1RN | 6.213 | 3.271 | 0.007 |
| ADAMDEC1 | 18.967 | 29.309 | 0.007 |
| SLC35F2 | 2.053 | 1.557 | 0.007 |
| IARS | 5.564 | 4.582 | 0.008 |
| ALDH1A2 | 5.482 | 2.868 | 0.008 |
| LOC729660 | 0.276 | 0.381 | 0.008 |
| IL18BP | 4.063 | 3.161 | 0.009 |
| ADAMDEC1 | 11.398 | 18.145 | 0.009 |
| NKIRAS1 | 2.767 | 2.435 | 0.009 |
| LOC100130229 | 0.161 | 0.243 | 0.009 |
| METTL1 | 4.948 | 4.097 | 0.009 |
| MTHFD1 | 2.252 | 1.888 | 0.010 |
| M6PR | 2.498 | 2.224 | 0.010 |
| LOC729021 | 0.335 | 0.438 | 0.011 |
| DGKA | 0.423 | 0.502 | 0.011 |
| NES | 3.061 | 2.292 | 0.012 |
| C17orf58 | 2.472 | 2.066 | 0.012 |
| C14orf169 | 3.187 | 2.496 | 0.012 |
| ITGA4 | 0.408 | 0.316 | 0.012 |
|  | 2.169 | 1.577 | 0.013 |
|  | 0.248 | 0.472 | 0.013 |
| LOC728728 | 0.449 | 0.559 | 0.014 |
| C14orf109 | 5.794 | 4.744 | 0.014 |
| LOC100134634 | 0.380 | 0.482 | 0.015 |
| LOC642590 | 2.551 | 2.154 | 0.015 |
| SLMO1 | 3.191 | 2.634 | 0.016 |
| SLC7A1 | 3.333 | 2.761 | 0.016 |
| PTP4A2 | 0.382 | 0.485 | 0.016 |
| RAB13 | 3.673 | 4.719 | 0.017 |
| SLC35F5 | 2.849 | 2.476 | 0.018 |
| LOC100128326 | 0.294 | 0.396 | 0.018 |
| LOC730286 | 0.309 | 0.415 | 0.019 |
| KCNH3 | 0.383 | 0.448 | 0.019 |
| TPM2 | 4.855 | 3.587 | 0.019 |
| AHCY | 2.752 | 2.289 | 0.021 |
| DUSP22 | 0.358 | 0.435 | 0.021 |
| CYP1B1 | 2.549 | 3.544 | 0.021 |
| DHRS9 | 4.158 | 2.628 | 0.022 |
| SMS | 2.536 | 2.180 | 0.022 |
| PHF23 | 2.368 | 2.904 | 0.022 |
| SPHK1 | 2.241 | 1.762 | 0.024 |
| SKP1 | 0.394 | 0.350 | 0.024 |
| C1RL | 0.326 | 0.422 | 0.024 |
| PLA2G4B | 0.287 | 0.371 | 0.024 |
| IL1RN | 5.554 | 3.604 | 0.025 |
| DHDH | 2.888 | 1.972 | 0.025 |
| AKAP13 | 0.434 | 0.499 | 0.026 |
| LOC646301 | 0.404 | 0.516 | 0.026 |
| ASGR2 | 0.073 | 0.102 | 0.026 |
| LOC100134530 | 0.348 | 0.454 | 0.027 |
| CES1 | 0.204 | 0.439 | 0.027 |
| APBB3 | 0.412 | 0.553 | 0.027 |
| H1FX | 0.340 | 0.410 | 0.027 |
| YPEL3 | 0.262 | 0.389 | 0.027 |
| POMGNT1 | 2.457 | 2.270 | 0.027 |
| MAPK8IP3 | 0.342 | 0.415 | 0.028 |
| MTX2 | 2.235 | 1.982 | 0.028 |
| NCF1 | 0.121 | 0.232 | 0.029 |
| F3 | 2.365 | 1.672 | 0.029 |
| WDR12 | 2.213 | 1.959 | 0.029 |
| C12orf5 | 3.131 | 2.561 | 0.029 |
| FBXL18 | 2.321 | 2.600 | 0.029 |
| ST7 | 2.683 | 2.347 | 0.030 |
| ADD3 | 0.299 | 0.351 | 0.030 |
| AGTRAP | 0.367 | 0.464 | 0.030 |
| COL23A1 | 2.284 | 3.509 | 0.030 |
| SPRED1 | 6.139 | 5.002 | 0.030 |
| TMOD2 | 0.391 | 0.482 | 0.031 |
| PLXNA3 | 1.963 | 2.170 | 0.032 |
| SEPHS2 | 2.768 | 3.386 | 0.032 |
| NAAA | 0.328 | 0.285 | 0.032 |
| FAM100B | 0.456 | 0.552 | 0.032 |
| GOT1 | 3.816 | 3.429 | 0.033 |
| SMAP2 | 0.274 | 0.336 | 0.033 |
| BRD3 | 0.362 | 0.469 | 0.033 |
| PDIA6 | 2.187 | 1.924 | 0.034 |
| PHF21A | 0.456 | 0.524 | 0.034 |
| PGS1 | 0.401 | 0.466 | 0.034 |
| RASGRP4 | 0.371 | 0.418 | 0.035 |
| ENOPH1 | 2.124 | 1.873 | 0.035 |
| TXNDC14 | 2.229 | 2.028 | 0.035 |
| WDR8 | 0.366 | 0.438 | 0.035 |
| C14orf138 | 0.508 | 0.463 | 0.035 |
| SLC35F2 | 3.281 | 2.445 | 0.036 |
| ALDH2 | 0.399 | 0.530 | 0.036 |
| CDK4 | 2.762 | 2.458 | 0.037 |
| CXCL10 | 0.367 | 0.264 | 0.037 |
| ANKRD57 | 1.767 | 2.268 | 0.038 |
| CCRL2 | 3.101 | 2.188 | 0.038 |
| HSPA1B | 3.745 | 5.222 | 0.038 |
| RBP1 | 1.816 | 2.434 | 0.038 |
| PTGFRN | 2.047 | 2.729 | 0.039 |
| NAAA | 0.393 | 0.349 | 0.039 |
| TMEM99 | 2.495 | 2.151 | 0.039 |
| C2orf7 | 2.442 | 2.155 | 0.039 |
| MIR155HG | 2.413 | 1.525 | 0.039 |
| EIF3G | 0.423 | 0.481 | 0.039 |
| NAAA | 0.365 | 0.322 | 0.040 |
| GATM | 4.276 | 3.222 | 0.040 |
| PNPLA2 | 0.342 | 0.405 | 0.040 |
| C16orf33 | 2.648 | 2.135 | 0.041 |
| ST3GAL6 | 2.561 | 2.223 | 0.041 |
| CYCSL1 | 2.288 | 1.904 | 0.041 |
| ABCA1 | 6.217 | 4.296 | 0.041 |
| NRG1 | 0.182 | 0.264 | 0.042 |
| MYO1F | 0.432 | 0.509 | 0.043 |
| GLRX3 | 2.652 | 2.323 | 0.043 |
| FMNL2 | 4.482 | 3.620 | 0.044 |
| NCF1B | 0.262 | 0.371 | 0.044 |
| FAM26F | 0.175 | 0.148 | 0.045 |
| GEM | 3.237 | 2.708 | 0.045 |
| HRASLS3 | 0.260 | 0.167 | 0.045 |
| PPAT | 2.083 | 1.875 | 0.045 |
| NUAK2 | 0.376 | 0.482 | 0.045 |
| SEMA4A | 0.298 | 0.412 | 0.045 |
| LOC642590 | 2.524 | 2.202 | 0.045 |
| PPM1H | 3.485 | 2.668 | 0.046 |
| OLFML3 | 2.258 | 1.675 | 0.047 |
| TPPP3 | 0.259 | 0.314 | 0.047 |
| SLC43A3 | 2.702 | 2.334 | 0.048 |
| PAICS | 2.709 | 2.306 | 0.048 |
| INF2 | 2.329 | 2.670 | 0.048 |
| LOC100131294 | 1.777 | 2.045 | 0.048 |
| LOC649555 | 2.444 | 2.107 | 0.049 |
| MKNK2 | 0.393 | 0.444 | 0.049 |

B.     163 significant genes with p<0.05 when comparing macrophage/monocyte expression between those with (SLE-A) or without atherosclerosis (SLE-NA).

| Gene | SLE-A Fold Change | SLE-NA fold change | p-value SLE-A vs SLE-NA |
| --- | --- | --- | --- |
| ALDH1A2 | 6.329 | 2.976 | 0.001 |
| CDKN1C | 0.223 | 0.155 | 0.002 |
| ADHFE1 | 0.378 | 0.510 | 0.002 |
| RHEB | 2.171 | 1.823 | 0.003 |
| ACAP1 | 0.373 | 0.529 | 0.003 |
| C10orf32 | 0.670 | 0.506 | 0.003 |
| TUBB1 | 0.289 | 0.625 | 0.003 |
| SLMO1 | 3.430 | 2.562 | 0.004 |
| LOC652616 | 0.167 | 0.257 | 0.004 |
| LOC729660 | 0.251 | 0.435 | 0.004 |
| SNX24 | 2.290 | 1.954 | 0.005 |
| EPB41L2 | 2.893 | 2.189 | 0.005 |
| SMARCD3 | 0.114 | 0.145 | 0.005 |
| HMGCL | 2.408 | 2.952 | 0.005 |
| LOC440957 | 3.035 | 2.347 | 0.006 |
| SPRED1 | 6.627 | 4.775 | 0.007 |
| KCNH3 | 0.366 | 0.468 | 0.007 |
| SERPINA1 | 0.106 | 0.204 | 0.007 |
| L3MBTL3 | 0.419 | 0.367 | 0.007 |
| PCBD1 | 2.185 | 2.747 | 0.007 |
| ADD3 | 0.273 | 0.344 | 0.007 |
| NCOA1 | 0.414 | 0.514 | 0.007 |
| CDC42EP2 | 0.478 | 0.364 | 0.008 |
| LOC728457 | 0.394 | 0.575 | 0.008 |
| SFRS17A | 0.403 | 0.505 | 0.008 |
|  | 0.237 | 0.501 | 0.008 |
| MMP7 | 74.944 | 113.515 | 0.008 |
| ETNK1 | 0.331 | 0.272 | 0.008 |
| MAPK8IP3 | 0.326 | 0.446 | 0.008 |
| HBB | 0.239 | 0.632 | 0.008 |
| TXNDC14 | 2.268 | 1.924 | 0.008 |
| LOC728728 | 0.425 | 0.597 | 0.009 |
| LOC100134634 | 0.380 | 0.553 | 0.009 |
| DSTN | 2.885 | 2.423 | 0.009 |
| LOC100128326 | 0.273 | 0.444 | 0.010 |
| SERPINA1 | 0.113 | 0.219 | 0.011 |
| LOC100130914 | 0.292 | 0.467 | 0.011 |
| SEL1L3 | 1.728 | 2.899 | 0.011 |
| DGKA | 0.385 | 0.507 | 0.011 |
| ABHD6 | 2.593 | 2.014 | 0.011 |
| LOC729021 | 0.323 | 0.487 | 0.012 |
| YPEL3 | 0.240 | 0.394 | 0.012 |
| C1orf122 | 2.263 | 2.948 | 0.012 |
| ST3GAL6 | 2.602 | 2.191 | 0.012 |
| C15orf58 | 1.991 | 2.507 | 0.012 |
| FPR1 | 0.050 | 0.103 | 0.013 |
| CCL2 | 21.193 | 8.732 | 0.013 |
| CRIP1 | 0.139 | 0.247 | 0.013 |
| C14orf109 | 6.253 | 4.908 | 0.014 |
| WDR8 | 0.350 | 0.416 | 0.014 |
| TNFRSF11A | 2.278 | 1.708 | 0.014 |
| OSBPL1A | 3.095 | 2.249 | 0.015 |
| SLC35F5 | 3.009 | 2.449 | 0.015 |
| SLC25A19 | 2.928 | 3.882 | 0.016 |
| DDX21 | 0.495 | 0.381 | 0.017 |
| LOC100130511 | 2.263 | 1.921 | 0.017 |
| ME2 | 2.074 | 1.663 | 0.017 |
| PPBP | 0.820 | 4.436 | 0.017 |
| APOBEC3F | 0.460 | 0.575 | 0.017 |
| FAM195A | 2.676 | 2.203 | 0.018 |
| M6PR | 2.566 | 2.193 | 0.019 |
| MRPL50 | 2.508 | 2.088 | 0.019 |
| MERTK | 2.725 | 2.209 | 0.019 |
| DHDH | 3.570 | 2.089 | 0.019 |
| IL1RN | 6.594 | 3.557 | 0.020 |
| CTSL2 | 3.584 | 2.397 | 0.020 |
| SLC43A3 | 2.577 | 2.075 | 0.021 |
| EPAS1 | 17.170 | 13.183 | 0.021 |
| HRASLS3 | 0.304 | 0.153 | 0.022 |
| FBXO5 | 1.981 | 1.411 | 0.022 |
| TMEM99 | 2.744 | 2.182 | 0.022 |
| DUSP1 | 0.057 | 0.039 | 0.022 |
| AOAH | 0.217 | 0.313 | 0.023 |
| ABP1 | 1.662 | 2.398 | 0.023 |
| PTP4A2 | 0.360 | 0.504 | 0.024 |
| CYCSL1 | 2.396 | 1.922 | 0.024 |
| CADM1 | 5.996 | 3.350 | 0.024 |
| MEGF9 | 0.174 | 0.223 | 0.024 |
| NR4A2 | 0.045 | 0.034 | 0.024 |
| IL18BP | 4.418 | 3.291 | 0.024 |
| CSF3R | 0.174 | 0.245 | 0.024 |
| NKIRAS1 | 2.802 | 2.425 | 0.025 |
| LIPN | 0.459 | 0.363 | 0.025 |
| DISP1 | 2.654 | 2.136 | 0.025 |
| HLA-F | 0.274 | 0.376 | 0.025 |
| LOC730286 | 0.312 | 0.473 | 0.025 |
| FDX1L | 2.671 | 2.289 | 0.026 |
| MAPKAP1 | 2.651 | 2.148 | 0.026 |
| SERPINE1 | 2.000 | 1.572 | 0.027 |
| AGTPBP1 | 0.229 | 0.275 | 0.027 |
| COTL1 | 0.378 | 0.472 | 0.027 |
| FBXL18 | 2.147 | 2.577 | 0.028 |
|  | 2.402 | 1.661 | 0.029 |
| C19orf22 | 0.403 | 0.493 | 0.029 |
| GPR109B | 0.357 | 0.272 | 0.029 |
| AHCY | 2.846 | 2.287 | 0.029 |
| B3GNT2 | 4.246 | 3.155 | 0.029 |
| CRTC2 | 0.496 | 0.578 | 0.030 |
| PPP1R15A | 0.243 | 0.180 | 0.030 |
| LOC401357 | 0.295 | 0.436 | 0.031 |
| NCOA1 | 0.412 | 0.472 | 0.031 |
| LOC649555 | 2.578 | 2.086 | 0.031 |
| DOPEY2 | 2.617 | 2.049 | 0.031 |
| PTCRA | 2.384 | 3.663 | 0.031 |
| FAM100B | 0.438 | 0.539 | 0.031 |
| HEBP1 | 2.307 | 1.886 | 0.033 |
| C12orf5 | 3.276 | 2.659 | 0.033 |
| UQCRQ | 2.277 | 2.046 | 0.034 |
| CDK5RAP3 | 0.443 | 0.522 | 0.034 |
| ITGA4 | 0.408 | 0.299 | 0.034 |
| LOC642590 | 2.694 | 2.200 | 0.034 |
| MERTK | 3.753 | 2.864 | 0.035 |
| PLXNA3 | 1.926 | 2.225 | 0.035 |
| AK3 | 2.243 | 2.599 | 0.036 |
| NUAK2 | 0.271 | 0.416 | 0.036 |
| SLC24A4 | 0.308 | 0.263 | 0.036 |
| CLEC7A | 0.296 | 0.182 | 0.036 |
| RRM2 | 2.585 | 2.013 | 0.038 |
| C17orf58 | 2.383 | 2.004 | 0.038 |
| PCBD1 | 3.335 | 3.941 | 0.038 |
|  | 0.352 | 0.415 | 0.038 |
| ANXA2 | 3.748 | 2.938 | 0.039 |
| LOC100134530 | 0.354 | 0.509 | 0.039 |
| GIMAP4 | 0.392 | 0.593 | 0.039 |
| NCF1B | 0.260 | 0.340 | 0.039 |
| RAB13 | 3.383 | 4.322 | 0.039 |
| CYP1B1 | 2.131 | 3.296 | 0.040 |
| SLC43A3 | 2.566 | 2.209 | 0.040 |
| DHRS9 | 5.121 | 3.319 | 0.040 |
| CTDSPL | 2.786 | 3.943 | 0.040 |
| SNX24 | 4.517 | 3.378 | 0.041 |
| PLEKHB2 | 2.428 | 1.988 | 0.041 |
| PLD1 | 2.152 | 2.613 | 0.042 |
| ING3 | 0.369 | 0.305 | 0.042 |
| TKT | 0.418 | 0.521 | 0.042 |
|  | 0.424 | 0.542 | 0.042 |
| FAM20C | 4.374 | 5.861 | 0.042 |
| CENPE | 2.170 | 1.766 | 0.043 |
| QDPR | 2.379 | 2.061 | 0.043 |
| F5 | 0.127 | 0.154 | 0.043 |
| C1RL | 0.311 | 0.408 | 0.044 |
| C3orf64 | 2.949 | 2.421 | 0.044 |
| BTN3A3 | 0.428 | 0.540 | 0.044 |
| FRMD4A | 8.419 | 6.464 | 0.045 |
| TLR8 | 0.276 | 0.392 | 0.045 |
| NCF1 | 0.104 | 0.181 | 0.045 |
| GPR34 | 8.293 | 5.458 | 0.046 |
| GLRX3 | 2.700 | 2.326 | 0.046 |
| LOC100128098 | 0.375 | 0.552 | 0.046 |
| TSPAN33 | 2.360 | 3.622 | 0.047 |
| CCNA2 | 4.501 | 3.216 | 0.047 |
| RPGRIP1 | 0.535 | 0.442 | 0.048 |
| PGS1 | 0.387 | 0.470 | 0.048 |
| TRABD | 0.431 | 0.513 | 0.048 |
| FASTKD1 | 2.074 | 1.783 | 0.048 |
| KLF6 | 0.358 | 0.253 | 0.048 |
| TMOD2 | 0.381 | 0.498 | 0.048 |
| GPBAR1 | 0.118 | 0.086 | 0.049 |
| GPR82 | 2.289 | 1.855 | 0.049 |
| CLEC7A | 0.263 | 0.203 | 0.049 |
| TCF7L2 | 0.455 | 0.371 | 0.049 |
| OLFML3 | 2.476 | 1.670 | 0.050 |
| PHF21A | 0.426 | 0.516 | 0.050 |

C. 74 (2.4%) genes with p<0.05 when comparing macrophage/monocyte expression between control individuals with (Ctrl-A) or without (Ctrl-NA) atherosclerosis.

| Gene | Ctrl-A Fold Change | Ctrl-NA Fold Change | p-value Ctrl-A vs Ctrl-NA |
| --- | --- | --- | --- |
| CAMKK2 | 0.331 | 0.503 | 0.001 |
| CITED4 | 0.511 | 0.300 | 0.001 |
| ADAMDEC1 | 18.697 | 32.880 | 0.001 |
| SLC35A2 | 2.898 | 2.334 | 0.002 |
| DKFZp761E198 | 0.456 | 0.601 | 0.003 |
| SLC35F2 | 2.358 | 1.596 | 0.004 |
| SMAD3 | 0.226 | 0.161 | 0.007 |
| SLC24A4 | 0.292 | 0.217 | 0.007 |
| FAM119B | 3.531 | 2.720 | 0.007 |
| ADAMDEC1 | 11.418 | 19.844 | 0.008 |
| LOC143666 | 0.390 | 0.549 | 0.008 |
| HSP90B1 | 2.227 | 1.789 | 0.008 |
| C14orf169 | 3.229 | 2.216 | 0.009 |
| NES | 3.318 | 1.909 | 0.010 |
| NAAA | 0.372 | 0.296 | 0.010 |
| METTL1 | 5.003 | 3.741 | 0.011 |
| SLC9A3R1 | 0.326 | 0.250 | 0.011 |
| PAPSS2 | 2.241 | 3.081 | 0.011 |
| TNS3 | 5.548 | 4.444 | 0.012 |
| LOC100130229 | 0.159 | 0.267 | 0.012 |
| NR4A2 | 0.037 | 0.049 | 0.013 |
| ADCK2 | 4.207 | 3.076 | 0.016 |
| GCH1 | 0.324 | 0.244 | 0.016 |
| LOC390251 | 2.323 | 1.624 | 0.016 |
| JUP | 0.587 | 0.420 | 0.017 |
| ESYT1 | 2.273 | 1.779 | 0.017 |
| MPEG1 | 0.242 | 0.341 | 0.017 |
| LOC100133866 | 3.503 | 5.934 | 0.017 |
| MAGED1 | 5.197 | 3.761 | 0.018 |
| NCRNA00120 | 0.304 | 0.449 | 0.018 |
| EVI2A | 0.314 | 0.453 | 0.019 |
| EYA3 | 0.408 | 0.371 | 0.020 |
| OTUD1 | 0.202 | 0.284 | 0.022 |
| CYTL1 | 2.837 | 1.911 | 0.022 |
| SLC35F2 | 3.918 | 2.570 | 0.023 |
| NAAA | 0.394 | 0.323 | 0.023 |
| FUT4 | 0.514 | 0.377 | 0.024 |
| LOC651309 | 0.280 | 0.446 | 0.026 |
| PDIA6 | 2.242 | 1.803 | 0.026 |
| FAM26F | 0.187 | 0.149 | 0.026 |
| FAS | 0.529 | 0.401 | 0.029 |
| VIPR1 | 0.169 | 0.136 | 0.030 |
| MTP18 | 2.169 | 1.860 | 0.030 |
| MRPL37 | 2.388 | 1.969 | 0.032 |
| SYTL1 | 0.323 | 0.403 | 0.032 |
| ACOT7 | 10.489 | 7.246 | 0.032 |
| ST7 | 2.822 | 2.256 | 0.034 |
| GABBR1 | 0.239 | 0.289 | 0.035 |
| CYTIP | 0.329 | 0.417 | 0.036 |
| SLC17A9 | 2.276 | 1.731 | 0.036 |
| TTC32 | 0.332 | 0.446 | 0.037 |
| SNORA28 | 0.276 | 0.371 | 0.037 |
| FCAR | 0.166 | 0.251 | 0.038 |
| COL23A1 | 2.322 | 3.964 | 0.038 |
| LOC653157 | 0.402 | 0.570 | 0.038 |
| PPAT | 2.226 | 1.894 | 0.039 |
| TMEM118 | 2.447 | 2.051 | 0.040 |
| ECHS1 | 1.888 | 1.669 | 0.041 |
| FAM39DP | 0.409 | 0.487 | 0.041 |
| TRIM22 | 0.313 | 0.254 | 0.041 |
| ZNF200 | 1.937 | 2.224 | 0.042 |
| JUND | 0.463 | 0.546 | 0.042 |
| CCRL2 | 2.880 | 1.987 | 0.042 |
| TMED3 | 3.714 | 3.190 | 0.042 |
| SLC7A1 | 3.552 | 2.880 | 0.044 |
| ZBTB16 | 0.406 | 0.516 | 0.044 |
| DUSP1 | 0.034 | 0.066 | 0.045 |
| C19orf38 | 0.480 | 0.404 | 0.045 |
| APOO | 4.562 | 3.259 | 0.045 |
| MOSPD1 | 2.616 | 1.974 | 0.045 |
| UCHL1 | 19.721 | 8.966 | 0.048 |
| LOC643332 | 0.258 | 0.367 | 0.048 |
| VSTM1 | 0.193 | 0.336 | 0.049 |
| ST7 | 2.852 | 2.380 | 0.049 |

D.    Overlap between gene lists in A-C with previously identified 344 gene signature

| Probes overlapping 344 gene signature | | |
| --- | --- | --- |
| A vs NA | SLE-A vs SLE-NA | Ctrl-A vs Ctrl-NA |
| 11 | 22 | 3 |
|  |  |  |
| ADAMDEC1 | ADAMDEC1 | NAMPT |
| DKFZp761E198 | C14orf138 | SLC35F2 |
| DGKA | CLEC7A | ST7 |
| DUSP1 | CSF3R |  |
| LOC642590 | DGKA |  |
| LOC729021 | DUSP1 |  |
| MAGED1 | F5 |  |
| NAAA | GPR109B |  |
| SLC24A4 | IL1RN |  |
| SLC35F2 | LOC729021 |  |
| ST3GAL6 | MERTK |  |
|  | NAMPT |  |
|  | NCF1 |  |
|  | NCOA1 |  |
|  | PCBD1 |  |
|  | NAAA |  |
|  | NCF1 |  |
|  | SERPINA1 |  |
|  | SLC43A3 |  |
|  | SMARCD3 |  |
|  | SNX24 |  |
|  | ST3GAL6 |  |
