# Supplementary material for: Inflammatory expression profiles in monocyte-to-macrophage differentiation in patients with systemic lupus erythematosus and relationship with atherosclerosis
Source: Arthritis Res Ther. 2014 Jul 10;16(4):R147. doi: 10.1186/ar4609 (PMC4227297; doi:10.1186/ar4609)

**Supplemental Figure 3.** Gene networks. Significant gene interaction networks were determined using pathway analysis was performed using Ingenuity Pathways Analysis (IPA) (Ingenuity Systems, Redwood City, CA) software and interactions between members of genes identified in GO analysis from the PANTHER database are depicted. Shaded genes represent those from the differentially expressed gene list and those which are not shaded are other predicted members of the pathway which the literature suggests are implicated in the biological process and interact with the genes in the pathway.

1. Apoptosis


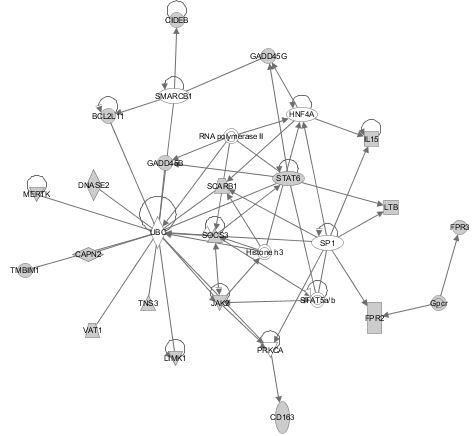


1. Carbohydrate metabolism, network 1


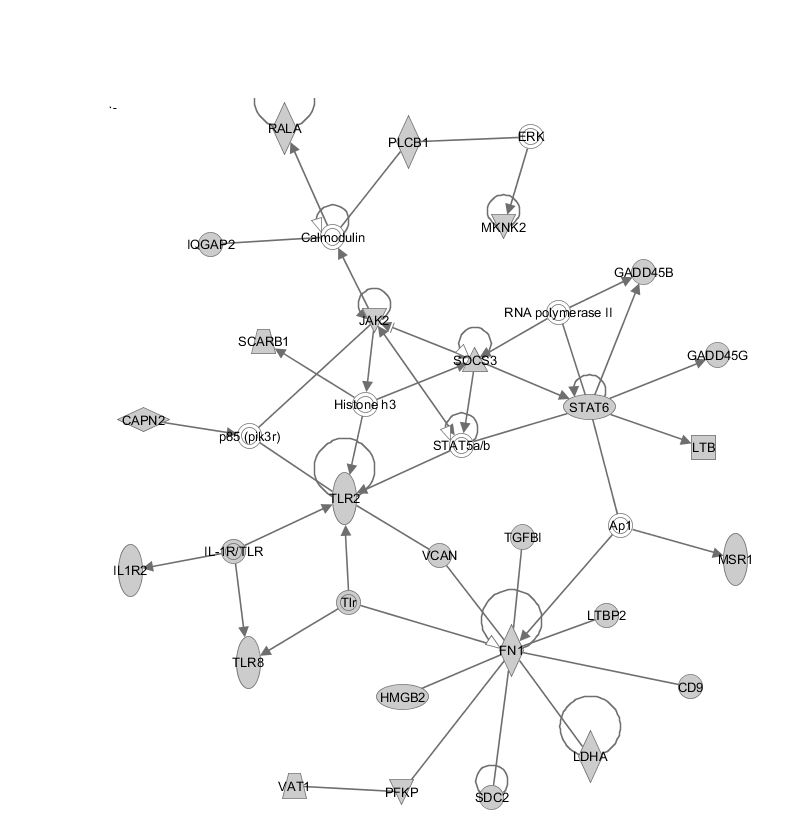


1. Carbohydrate metabolism, network 2


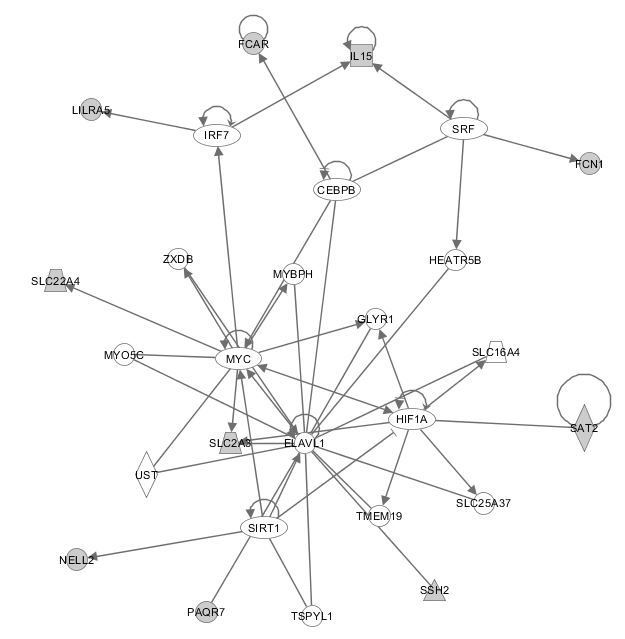


1. Carbohydrate metabolism, network 3


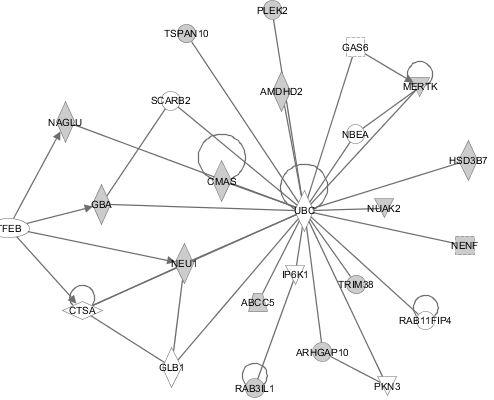


1. Immune system process, network 1


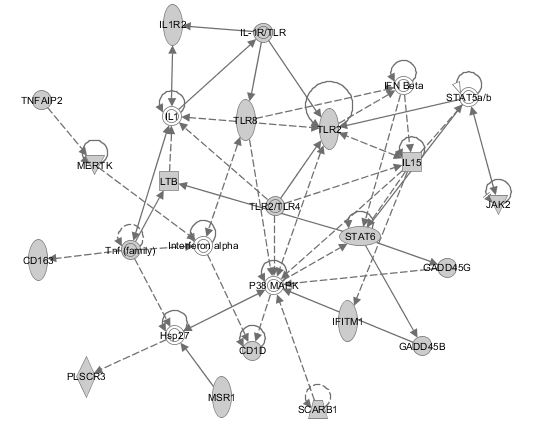


1. Immune system process, network 2


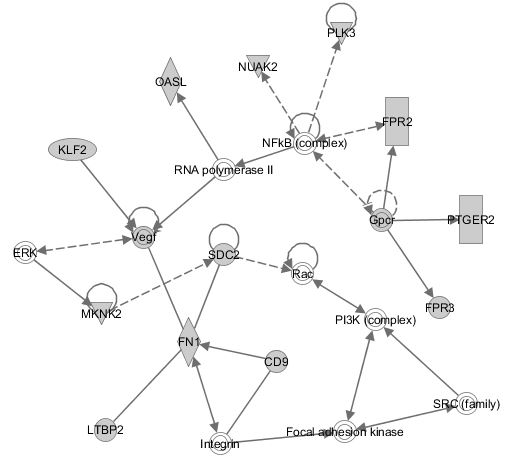


1. Lipid metabolism, network 1


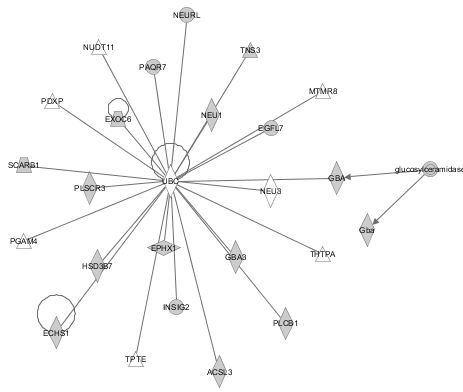


1. Lipid metabolism, network 2


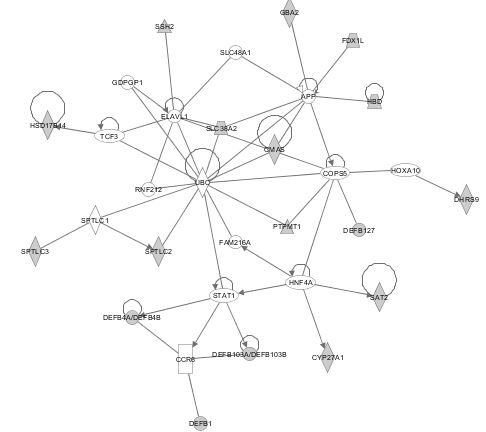


1. Signal transduction, network 1


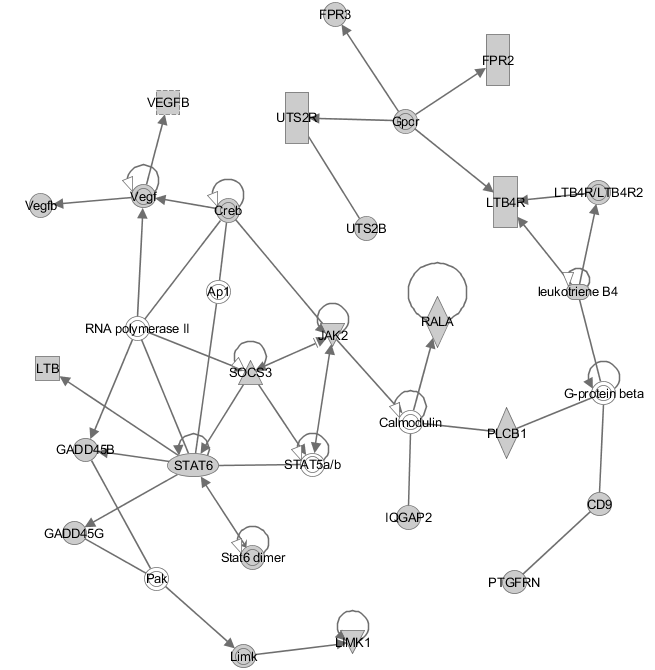


1. Signal transduction, network 2


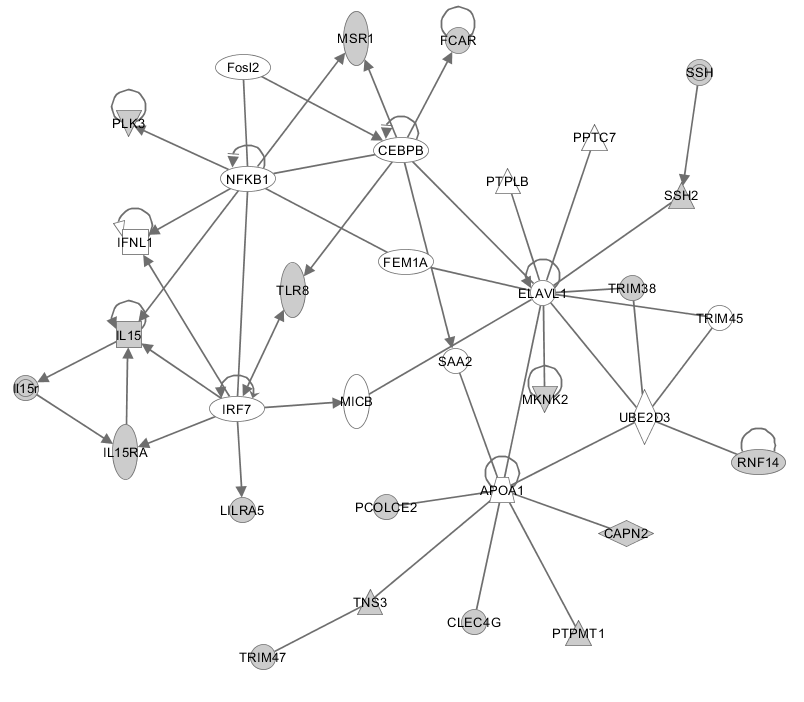

Supplement: Additional file 4 — Gene networks. Significant gene interaction networks were determined by using pathway analysis performed by using Ingenuity Pathways Analysis (IPA). GO, gene ontology; PANTHER, Protein Analysis Through Evolutionary Relationships. [file ar4609-S4.docx]
