# Supplementary material for: Inflammatory expression profiles in monocyte-to-macrophage differentiation in patients with systemic lupus erythematosus and relationship with atherosclerosis
Source: Arthritis Res Ther. 2014 Jul 10;16(4):R147. doi: 10.1186/ar4609 (PMC4227297; doi:10.1186/ar4609)

**Supplemental Figure 2**. Heatmaps for GO term gene members. All heatmaps were generated using CLUSTER and TREEVIEW using hierarchical clustering based on both sample and gene expression similarity. On each tree dendogram subjects are listed by study ID and group identity. .caa indicates an SLE patient with atherosclerosis, .can indicates an SLE patient without atherosclerosis, .coa indicates a control individual with atherosclerosis and .con indicates a control individual without atherosclerosis

1. Apoptosis, downregulated genes


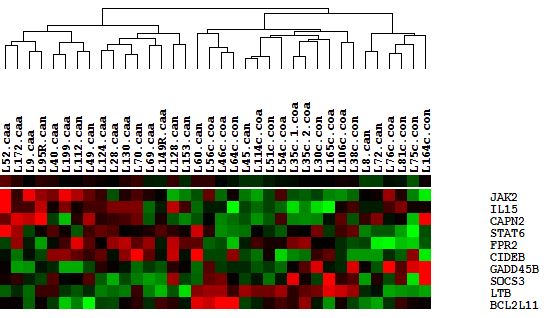


1. Apoptosis, upregulated genes


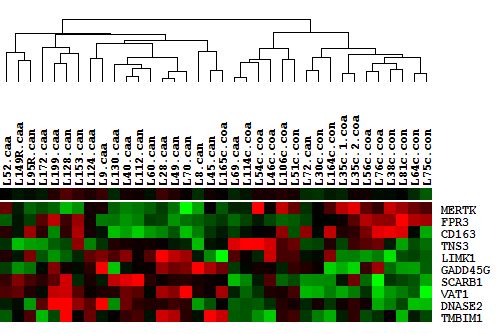


1. Carbohydrate metabolism, downregulated genes


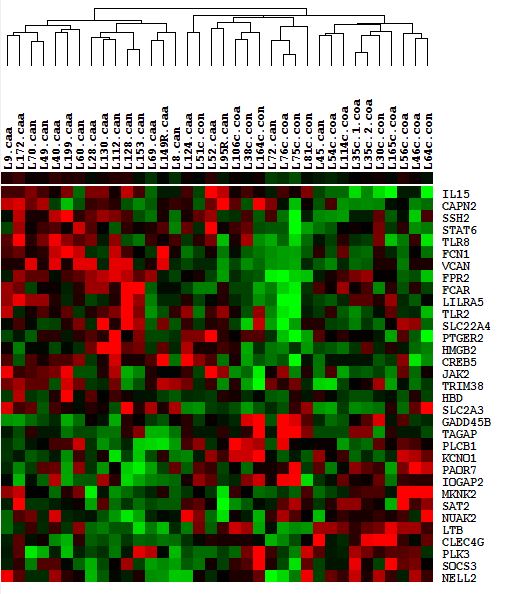


1. Carbohydrate metabolism, upregulated genes


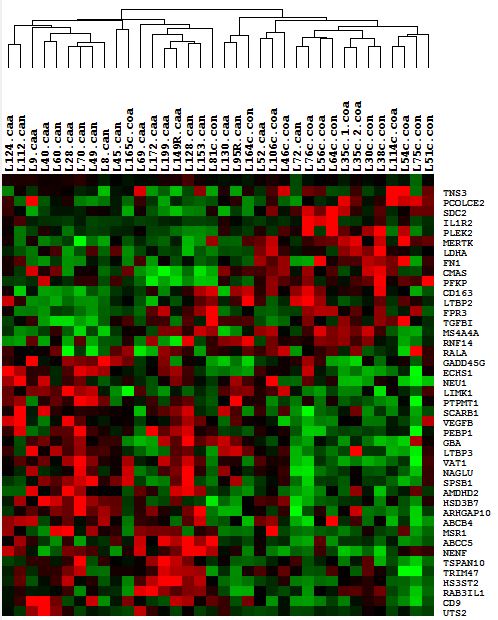


1. Immune system process, downregulated genes


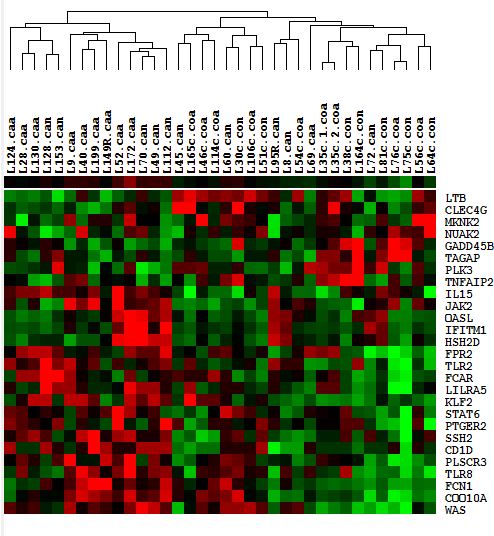


1. Immune system process, upregulated genes


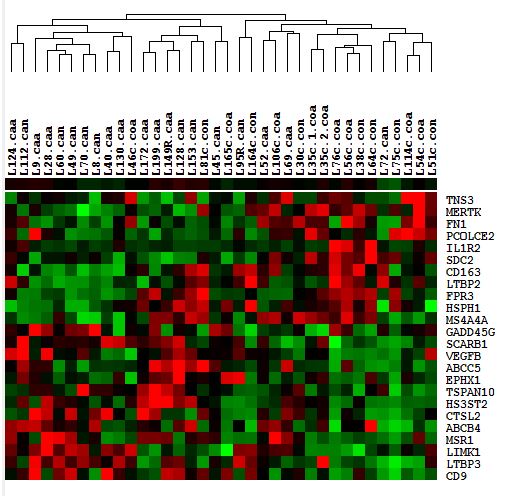


1. Lipid metabolism, downregulated genes


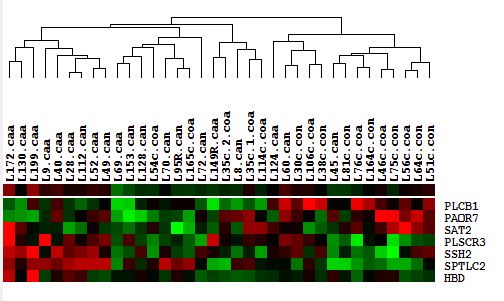


1. Lipid metabolism, upregulated genes


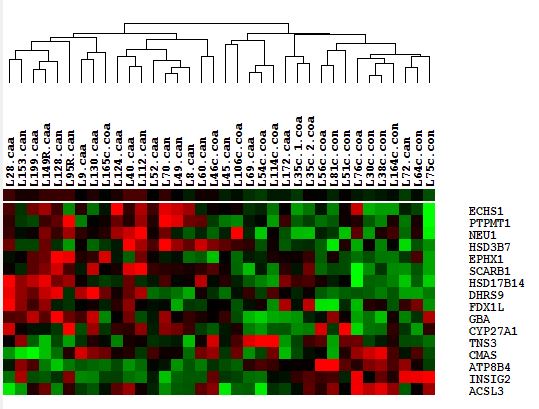


1. Signal transduction, downregulated genes (also see Figure 1c)
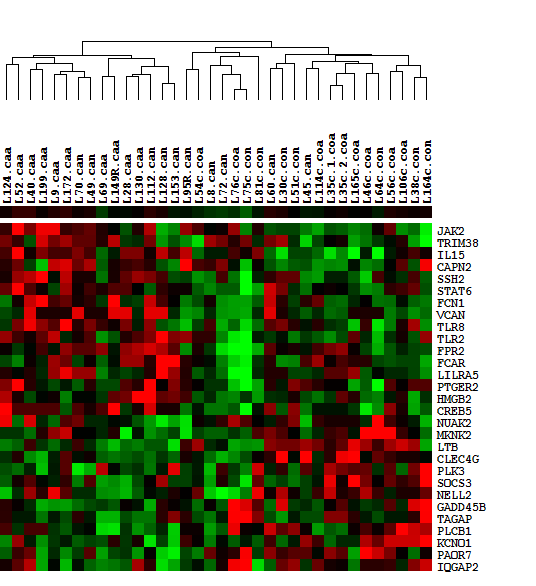

2. Signal transduction, upregulated genes


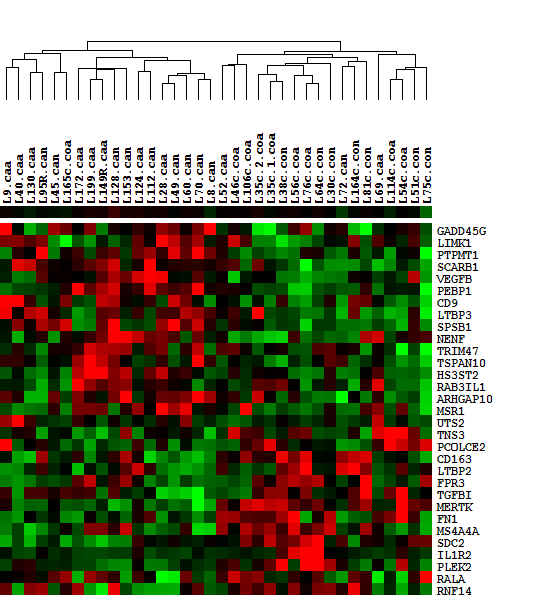

Supplement: Additional file 5 — Gene ontology (GO) term breakdowns. Heatmaps derived from genes representing significant GO terms for all systemic lupus erythematosus (SLE) cases and controls are shown. [file ar4609-S5.docx]
