# Supplementary material for: Inflammatory expression profiles in monocyte-to-macrophage differentiation in patients with systemic lupus erythematosus and relationship with atherosclerosis
Source: Arthritis Res Ther. 2014 Jul 10;16(4):R147. doi: 10.1186/ar4609 (PMC4227297; doi:10.1186/ar4609)
Supplement: Additional file 6 — Three hundred forty-four-gene atherosclerosis signature. Gene list for previously defined 344-gene atherosclerosis gene signature is shown. [file ar4609-S6.docx]

**Supplemental Table 3.** Previously defined 344 gene atherosclerosis gene signature

| **Gene Symbol** | **Gene Name** |
| --- | --- |
| FCGR2A | Fc fragment of IgG, low affinity IIa, receptor (CD32) |
|  | potassium inwardly-rectifying channel, subfamily J, member 2 |
| KCNJ2 |  |
| STX3 | syntaxin 3 |
| TLR8 | toll-like receptor 8 |
| ACSL1 | acyl-CoA synthetase long-chain family member 1 |
| CLEC4E | C-type lectin domain family 4, member E |
| TLR8 | toll-like receptor 8 |
| TLR4 | toll-like receptor 4 |
| TM6SF1 | transmembrane 6 superfamily member 1 |
| CASP4 | caspase 4, apoptosis-related cysteine peptidase |
| NAMPT | nicotinamide phosphoribosyltransferase |
| FPRL1 | formyl peptide receptor 2 |
| LOC399744 | hypothetical LOC399744 |
| QPCT | glutaminyl-peptide cyclotransferase |
| MCL1 | myeloid cell leukemia sequence 1 (BCL2-related) |
| P2RY13 | purinergic receptor P2Y, G-protein coupled, 13 |
| TNFSF14 | tumor necrosis factor (ligand) superfamily, member 14 |
| IL1R2 | interleukin 1 receptor, type II |
| TGFA | transforming growth factor, alpha |
| LOC730820 | NA |
| ABLIM1 | actin binding LIM protein 1 |
| GK | glycerol kinase |
| GPR97 | G protein-coupled receptor 97 |
| VNN2 | vanin 2 |
| STX11 | syntaxin 11 |
| CA4 | carbonic anhydrase IV |
| TLR2 | toll-like receptor 2 |
| MME | membrane metallo-endopeptidase |
| FLJ20273 | RNA binding motif protein 47 |
| RAB24 | RAB24, member RAS oncogene family |
| TNFRSF10B | tumor necrosis factor receptor superfamily, member 10b |
| IL1RN | interleukin 1 receptor antagonist |
| IL1B | interleukin 1, beta |
| FAM126B | family with sequence similarity 126, member B |
| TLR6 | toll-like receptor 6 |
| CPD | carboxypeptidase D |
| MGAM | maltase-glucoamylase (alpha-glucosidase) |
| ENTPD1 | ectonucleoside triphosphate diphosphohydrolase 1 |
| LRG1 | leucine-rich alpha-2-glycoprotein 1 |
| NFIL3 | nuclear factor, interleukin 3 regulated |
| FFAR2 | free fatty acid receptor 2 |
| SMARCD3 | SWI/SNF related, matrix associated, actin dependent regulator of chromatin, subfamily d, member 3 |
| ROPN1L | ropporin 1-like |
| LAMP2 | lysosomal-associated membrane protein 2 |
| FAIM3 | Fas apoptotic inhibitory molecule 3 |
| B4GALT5 | UDP-Gal:betaGlcNAc beta 1,4- galactosyltransferase, polypeptide 5 |
| MYBPC3 | myosin binding protein C, cardiac |
| EIF4E3 | eukaryotic translation initiation factor 4E family member 3 |
| CREB5 | cAMP responsive element binding protein 5 |
| LIMK2 | LIM domain kinase 2 |
| CSF2RB | colony stimulating factor 2 receptor, beta, low-affinity (granulocyte-macrophage) |
| LILRA5 | leukocyte immunoglobulin-like receptor, subfamily A (with TM domain), member 5 |
| MAPK14 | mitogen-activated protein kinase 14 |
| KCNJ15 | potassium inwardly-rectifying channel, subfamily J, member 15 |
| STEAP4 | STEAP family member 4 |
| NCF4 | neutrophil cytosolic factor 4, 40kDa |
| GNG10 | guanine nucleotide binding protein (G protein), gamma 10 |
| PILRA | paired immunoglobin-like type 2 receptor alpha |
| SIRPB1 | signal-regulatory protein beta 1 |
| CDC25B | cell division cycle 25 homolog B (S. pombe) |
| FRAT1 | frequently rearranged in advanced T-cell lymphomas |
| RAB24 | RAB24, member RAS oncogene family |
| CEACAM3 | carcinoembryonic antigen-related cell adhesion molecule 3 |
| NCF4 | neutrophil cytosolic factor 4, 40kDa |
| TLR1 | toll-like receptor 1 |
| CCDC64 | coiled-coil domain containing 64 |
| MEFV | Mediterranean fever |
| MOSC1 | MOCO sulphurase C-terminal domain containing 1 |
| SNAP23 | synaptosomal-associated protein, 23kDa |
| WDFY3 | WD repeat and FYVE domain containing 3 |
| GAB2 | GRB2-associated binding protein 2 |
| ABHD5 | abhydrolase domain containing 5 |
| EGLN1 | egl nine homolog 1 (C. elegans) |
| BEST1 | bestrophin 1 |
| PHC2 | polyhomeotic homolog 2 (Drosophila) |
| LOC339745 | speckle-type POZ protein-like |
| IL6R | interleukin 6 receptor |
| ZNF438 | zinc finger protein 438 |
| KBTBD7 | kelch repeat and BTB (POZ) domain containing 7 |
| FRAT1 | frequently rearranged in advanced T-cell lymphomas |
| PLXNC1 | plexin C1 |
| UBE2L6 | ubiquitin-conjugating enzyme E2L 6 |
| NTNG2 | netrin G2 |
| CD27 | CD27 molecule |
| RCN3 | reticulocalbin 3, EF-hand calcium binding domain |
| LOC729021 | hypothetical protein LOC729021 |
| NAMPT | nicotinamide phosphoribosyltransferase |
| SIPA1L2 | signal-induced proliferation-associated 1 like 2 |
| FAM53C | family with sequence similarity 53, member C |
| PIK3IP1 | phosphoinositide-3-kinase interacting protein 1 |
| REM2 | RAS (RAD and GEM)-like GTP binding 2 |
| HCG27 | HLA complex group 27 |
| GPR97 | G protein-coupled receptor 97 |
| CLEC7A | C-type lectin domain family 7, member A |
| MAG1 | 1-acylglycerol-3-phosphate O-acyltransferase 9 |
| MANSC1 | MANSC domain containing 1 |
| EMR3 | egf-like module containing, mucin-like, hormone receptor-like 3 |
| PELI1 | pellino homolog 1 (Drosophila) |
| MAP4K1 | mitogen-activated protein kinase kinase kinase kinase 1 |
| IL8RB | interleukin 8 receptor, beta |
| CSF3R | colony stimulating factor 3 receptor (granulocyte) |
| KIAA0125 | KIAA0125 |
| DYSF | dysferlin, limb girdle muscular dystrophy 2B (autosomal recessive) |
| CREB5 | cAMP responsive element binding protein 5 |
| HK2 | hexokinase 2 |
| KLHL2 | kelch-like 2, Mayven (Drosophila) |
| SELM | selenoprotein M |
| ST8SIA4 | ST8 alpha-N-acetyl-neuraminide alpha-2,8-sialyltransferase 4 |
| LDLRAP1 | low density lipoprotein receptor adaptor protein 1 |
| IL1R2 | interleukin 1 receptor, type II |
| PLAUR | plasminogen activator, urokinase receptor |
| RAB11FIP1 | RAB11 family interacting protein 1 (class I) |
| ELL | elongation factor RNA polymerase II |
| TSEN34 | tRNA splicing endonuclease 34 homolog (S. cerevisiae) |
| IDS | iduronate 2-sulfatase |
| OSM | oncostatin M |
| BTBD14A | NACC family member 2, BEN and BTB (POZ) domain containing |
| TIMP2 | TIMP metallopeptidase inhibitor 2 |
| DKFZp761E198 | DKFZp761E198 protein |
| NCF1 | neutrophil cytosolic factor 1 |
| MAPK14 | mitogen-activated protein kinase 14 |
| PLAUR | plasminogen activator, urokinase receptor |
| MOSPD2 | motile sperm domain containing 2 |
| NPL | N-acetylneuraminate pyruvate lyase (dihydrodipicolinate synthase) |
| DOCK5 | dedicator of cytokinesis 5 |
| IKIP | IKK interacting protein |
| ACOX1 | acyl-Coenzyme A oxidase 1, palmitoyl |
| HIATL1 | hippocampus abundant transcript-like 1 |
| ZDHHC18 | zinc finger, DHHC-type containing 18 |
| PILRA | paired immunoglobin-like type 2 receptor alpha |
| KIAA0319L | KIAA0319-like |
| RNF122 | ring finger protein 122 |
| CSF2RA | colony stimulating factor 2 receptor, alpha, low-affinity (granulocyte-macrophage) |
| IRAK3 | interleukin-1 receptor-associated kinase 3 |
| IL8RA | interleukin 8 receptor, alpha |
| ST3GAL4 | ST3 beta-galactoside alpha-2,3-sialyltransferase 4 |
| PTGS2 | prostaglandin-endoperoxide synthase 2 (prostaglandin G/H synthase and cyclooxygenase) |
| MAGED1 | melanoma antigen family D, 1 |
| FAM113B | family with sequence similarity 113, member B |
| ST6GALNAC2 | ST6 (alpha-N-acetyl-neuraminyl-2,3-beta-galactosyl-1,3)-N-acetylgalactosaminide alpha-2,6-sialyltransferase 2 |
| USP32 | ubiquitin specific peptidase 32 |
| TSHZ3 | teashirt zinc finger homeobox 3 |
| SLC22A4 | solute carrier family 22 (organic cation/ergothioneine transporter), member 4 |
| C3orf62 | chromosome 3 open reading frame 62 |
| C16orf57 | chromosome 16 open reading frame 57 |
| KIAA0746 | KIAA0746 protein |
| ATP6V0E2 | ATPase, H+ transporting V0 subunit e2 |
| TLR5 | toll-like receptor 5 |
| ABHD3 | abhydrolase domain containing 3 |
| ADM | adrenomedullin |
| HPCAL4 | hippocalcin like 4 |
| LST1 | leukocyte specific transcript 1 |
| ST8SIA4 | ST8 alpha-N-acetyl-neuraminide alpha-2,8-sialyltransferase 4 |
| STAT3 | signal transducer and activator of transcription 3 (acute-phase response factor) |
| DIRC2 | disrupted in renal carcinoma 2 |
| NADK | NAD kinase |
| DUSP1 | dual specificity phosphatase 1 |
| LAT2 | linker for activation of T cells family, member 2 |
| TNFSF14 | tumor necrosis factor (ligand) superfamily, member 14 |
| C14orf138 | chromosome 14 open reading frame 138 |
| ANXA3 | annexin A3 |
| PTPRCAP | protein tyrosine phosphatase, receptor type, C-associated protein |
| PLCG1 | phospholipase C, gamma 1 |
| C20orf3 | chromosome 20 open reading frame 3 |
| GPR109B | niacin receptor 2 |
| C9orf72 | chromosome 9 open reading frame 72 |
| DGKA | diacylglycerol kinase, alpha 80kDa |
| DENND3 | DENN/MADD domain containing 3 |
| REPS2 | RALBP1 associated Eps domain containing 2 |
| PYGL | phosphorylase, glycogen, liver |
| PTAFR | platelet-activating factor receptor |
| SKAP2 | src kinase associated phosphoprotein 2 |
| TMEM157 | family with sequence similarity 174, member A |
| FAM102A | family with sequence similarity 102, member A |
| LOC728014 | NA |
| MAK | male germ cell-associated kinase |
| GPR109A | niacin receptor 1 |
| CENTD3 | ArfGAP with RhoGAP domain, ankyrin repeat and PH domain 3 |
| BIN1 | bridging integrator 1 |
| PLXDC2 | plexin domain containing 2 |
| PANX2 | pannexin 2 |
| PSG3 | pregnancy specific beta-1-glycoprotein 3 |
| E2F3 | E2F transcription factor 3 |
| SIPA1L1 | signal-induced proliferation-associated 1 like 1 |
| SLC43A2 | solute carrier family 43, member 2 |
| MMP25 | matrix metallopeptidase 25 |
| INADL | InaD-like (Drosophila) |
| SLCO3A1 | solute carrier organic anion transporter family, member 3A1 |
| IRS2 | insulin receptor substrate 2 |
| BCL11B | B-cell CLL/lymphoma 11B (zinc finger protein) |
| RP2 | retinitis pigmentosa 2 (X-linked recessive) |
| EDG4 | lysophosphatidic acid receptor 2 |
| HSPA6 | heat shock 70kDa protein 6 (HSP70B') |
| IL6R | interleukin 6 receptor |
| TREM1 | triggering receptor expressed on myeloid cells 1 |
| CASP8 | caspase 8, apoptosis-related cysteine peptidase |
| SEC14L1 | SEC14-like 1 (S. cerevisiae) |
| GNAQ | guanine nucleotide binding protein (G protein), q polypeptide |
| Rgr | ral guanine nucleotide dissociation stimulator-like 4 |
| C10orf38 | family with sequence similarity 171, member A1 |
| TCF4 | transcription factor 4 |
| TTC27 | tetratricopeptide repeat domain 27 |
| IBRDC2 | ring finger protein 144B |
| EVA1 | myelin protein zero-like 2 |
| SPTAN1 | spectrin, alpha, non-erythrocytic 1 (alpha-fodrin) |
| CRISPLD2 | cysteine-rich secretory protein LCCL domain containing 2 |
| CXCL16 | chemokine (C-X-C motif) ligand 16 |
| TMCC3 | transmembrane and coiled-coil domain family 3 |
| RAB20 | RAB20, member RAS oncogene family |
| COBLL1 | COBL-like 1 |
| CDR2 | cerebellar degeneration-related protein 2, 62kDa |
| ECHDC2 | enoyl Coenzyme A hydratase domain containing 2 |
| SUSD3 | sushi domain containing 3 |
| GIMAP5 | GTPase, IMAP family member 5 |
| WSB1 | WD repeat and SOCS box-containing 1 |
| VNN3 | vanin 3 |
| C9orf164 | NA |
| RAB11FIP1 | RAB11 family interacting protein 1 (class I) |
| DPEP3 | dipeptidase 3 |
| BCAS4 | breast carcinoma amplified sequence 4 |
| AMD1 | adenosylmethionine decarboxylase 1 |
| ITM2C | integral membrane protein 2C |
| GPR177 | G protein-coupled receptor 177 |
| SASP | aspartic peptidase, retroviral-like 1 |
| SKAP1 | src kinase associated phosphoprotein 1 |
| CXXC5 | CXXC finger 5 |
| GNAI3 | guanine nucleotide binding protein (G protein), alpha inhibiting activity polypeptide 3 |
| LY9 | lymphocyte antigen 9 |
| RRAGD | Ras-related GTP binding D |
| SLC9A8 | solute carrier family 9 (sodium/hydrogen exchanger), member 8 |
| CCR7 | chemokine (C-C motif) receptor 7 |
| KLHL3 | kelch-like 3 (Drosophila) |
| ARRDC3 | arrestin domain containing 3 |
| C5AR1 | complement component 5a receptor 1 |
| FCGR3B | Fc fragment of IgG, low affinity IIIb, receptor (CD16b) |
| CLIC1 | chloride intracellular channel 1 |
| ITPR3 | inositol 1,4,5-triphosphate receptor, type 3 |
| MGC4093 | B9 protein domain 2 |
| C3orf34 | chromosome 3 open reading frame 34 |
| ATG16L2 | ATG16 autophagy related 16-like 2 (S. cerevisiae) |
| DKFZp434K1815 | leucine-rich repeats and WD repeat domain containing 1 |
| CUEDC1 | CUE domain containing 1 |
| RASGRP3 | RAS guanyl releasing protein 3 (calcium and DAG-regulated) |
| PSTPIP2 | proline-serine-threonine phosphatase interacting protein 2 |
| PROK2 | prokineticin 2 |
| PADI4 | peptidyl arginine deiminase, type IV |
| ZNF467 | zinc finger protein 467 |
| LOC340527 | NHS-like 2 |
| ARID3A | AT rich interactive domain 3A (BRIGHT-like) |
| SMPDL3A | sphingomyelin phosphodiesterase, acid-like 3A |
| DUSP6 | dual specificity phosphatase 6 |
| BLR1 | chemokine (C-X-C motif) receptor 5 |
| C9orf45 | NA |
| STXBP5 | syntaxin binding protein 5 (tomosyn) |
| C5orf32 | chromosome 5 open reading frame 32 |
| FCER2 | Fc fragment of IgE, low affinity II, receptor for (CD23) |
| PKN2 | protein kinase N2 |
| F5 | coagulation factor V (proaccelerin, labile factor) |
| MAN2C1 | mannosidase, alpha, class 2C, member 1 |
| HSDL2 | hydroxysteroid dehydrogenase like 2 |
| DOCK10 | dedicator of cytokinesis 10 |
| ITK | IL2-inducible T-cell kinase |
| ZAP70 | zeta-chain (TCR) associated protein kinase 70kDa |
| RNF141 | ring finger protein 141 |
| ID3 | inhibitor of DNA binding 3, dominant negative helix-loop-helix protein |
| NOTCH1 | Notch homolog 1, translocation-associated (Drosophila) |
| AFF3 | AF4/FMR2 family, member 3 |
| TIMP2 | TIMP metallopeptidase inhibitor 2 |
| DSC2 | desmocollin 2 |
| GBP2 | guanylate binding protein 2, interferon-inducible |
| WWC3 | WWC family member 3 |
| ALPL | alkaline phosphatase, liver/bone/kidney |
| CD79A | CD79a molecule, immunoglobulin-associated alpha |
| TMEM127 | transmembrane protein 127 |
| C11orf75 | chromosome 11 open reading frame 75 |
| CYP4F3 | cytochrome P450, family 4, subfamily F, polypeptide 3 |
| NLRP12 | NLR family, pyrin domain containing 12 |
| ADARB1 | adenosine deaminase, RNA-specific, B1 (RED1 homolog rat) |
| ERGIC1 | endoplasmic reticulum-golgi intermediate compartment (ERGIC) 1 |
| PARP9 | poly (ADP-ribose) polymerase family, member 9 |
| CD6 | CD6 molecule |
| PXN | paxillin |
| CEACAM1 | carcinoembryonic antigen-related cell adhesion molecule 1 (biliary glycoprotein) |
| PISD | phosphatidylserine decarboxylase |
| C13orf15 | chromosome 13 open reading frame 15 |
| LOC728776 | high-mobility group nucleosome binding domain 1 pseudogene |
| USP15 | ubiquitin specific peptidase 15 |
| PGLYRP1 | peptidoglycan recognition protein 1 |
| BLK | B lymphoid tyrosine kinase |
| RPS8 | ribosomal protein S8 |
| CDCA7L | cell division cycle associated 7-like |
| VPREB3 | pre-B lymphocyte 3 |
| CASP8 | caspase 8, apoptosis-related cysteine peptidase |
| TNFAIP2 | tumor necrosis factor, alpha-induced protein 2 |
| DHX34 | DEAH (Asp-Glu-Ala-His) box polypeptide 34 |
| USP10 | ubiquitin specific peptidase 10 |
| LOC201175 | SH3 domain containing 20 |
| S100A12 | S100 calcium binding protein A12 |
| TLE3 | transducin-like enhancer of split 3 (E(sp1) homolog, Drosophila) |
| ZNF101 | zinc finger protein 101 |
| LEF1 | lymphoid enhancer-binding factor 1 |
| OAZ2 | ornithine decarboxylase antizyme 2 |
| BCL2 | B-cell CLL/lymphoma 2 |
| CD302 | CD302 molecule |
| CD79A | CD79a molecule, immunoglobulin-associated alpha |
| HOOK1 | hook homolog 1 (Drosophila) |
| PGAM1 | phosphoglycerate mutase 1 (brain) |
| PDE7A | phosphodiesterase 7A |
| ST3GAL6 | ST3 beta-galactoside alpha-2,3-sialyltransferase 6 |
| PGD | phosphogluconate dehydrogenase |
| SOD2 | superoxide dismutase 2, mitochondrial |
| PACSIN1 | protein kinase C and casein kinase substrate in neurons 1 |
| FOS | FBJ murine osteosarcoma viral oncogene homolog |
| PTPRC | protein tyrosine phosphatase, receptor type, C |
| ZNF121 | zinc finger protein 121 |
| OKL38 | oxidative stress induced growth inhibitor 1 |
| TREML2 | triggering receptor expressed on myeloid cells-like 2 |
| PIK3AP1 | phosphoinositide-3-kinase adaptor protein 1 |
| CD3E | CD3e molecule, epsilon (CD3-TCR complex) |
| C16orf7 | chromosome 16 open reading frame 7 |
| EPHX2 | epoxide hydrolase 2, cytoplasmic |
| BCL11A | B-cell CLL/lymphoma 11A (zinc finger protein) |
| FBXL13 | F-box and leucine-rich repeat protein 13 |
| CDA | cytidine deaminase |
| UBN1 | ubinuclein 1 |
| PPAPDC1B | phosphatidic acid phosphatase type 2 domain containing 1B |
| S100P | S100 calcium binding protein P |
| HIST1H2AC | histone cluster 1, H2ac |
| BIRC3 | baculoviral IAP repeat-containing 3 |
| HSD17B11 | hydroxysteroid (17-beta) dehydrogenase 11 |
| LPPR2 | lipid phosphate phosphatase-related protein type 2 |
| KIAA0319L | KIAA0319-like |
| SRPK1 | SFRS protein kinase 1 |
| LHFPL2 | lipoma HMGIC fusion partner-like 2 |
| BST1 | bone marrow stromal cell antigen 1 |
| RNF24 | ring finger protein 24 |
| CFP | complement factor properdin |
| RPS5 | ribosomal protein S5 |
| SLC19A1 | solute carrier family 19 (folate transporter), member 1 |
| GBP1 | guanylate binding protein 1, interferon-inducible, 67kDa |
| ALDH16A1 | aldehyde dehydrogenase 16 family, member A1 |
| FKBP1A | FK506 binding protein 1A, 12kDa |
| CD93 | CD93 molecule |
| SESN1 | sestrin 1 |
