# Supplementary material for: Inflammatory expression profiles in monocyte-to-macrophage differentiation in patients with systemic lupus erythematosus and relationship with atherosclerosis
Source: Arthritis Res Ther. 2014 Jul 10;16(4):R147. doi: 10.1186/ar4609 (PMC4227297; doi:10.1186/ar4609)

**Supplementary Figure 3.** Heatmap demonstrating previously described 344 atherosclerosis gene signature in all SLE cases and controls. Subjects are listed by study ID and group identity. .caa indicates an SLE patient with atherosclerosis, .can indicates an SLE patient without atherosclerosis, .coa indicates a control individual with atherosclerosis and .con indicates a control individual without atherosclerosis


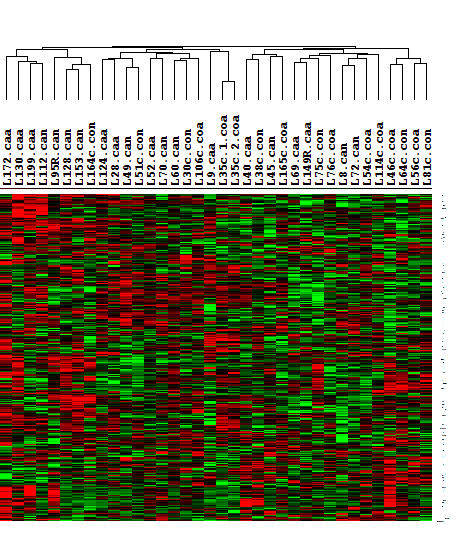

Supplement: Additional file 7 — Three hundred forty-four-gene atherosclerosis signature. Heatmap demonstrates previously described 344-atherosclerosis gene signature in all systemic lupus erythematosus (SLE) cases and controls. [file ar4609-S7.docx]
