# Supplementary material for: Inflammatory expression profiles in monocyte-to-macrophage differentiation in patients with systemic lupus erythematosus and relationship with atherosclerosis
Source: Arthritis Res Ther. 2014 Jul 10;16(4):R147. doi: 10.1186/ar4609 (PMC4227297; doi:10.1186/ar4609)
Supplement: Additional file 8 — Consensus clustering. Resampling-based consensus clustering analysis for 344-gene atherosclerosis signature is shown. [file ar4609-S8.docx]

**Supplemental Figure 4**. Resampling-based consensus clustering analysis for 344 gene atherosclerosis signature

1. Cumulative distribution functions (CDF) of the consensus matrix at each number of clusters (*K*)


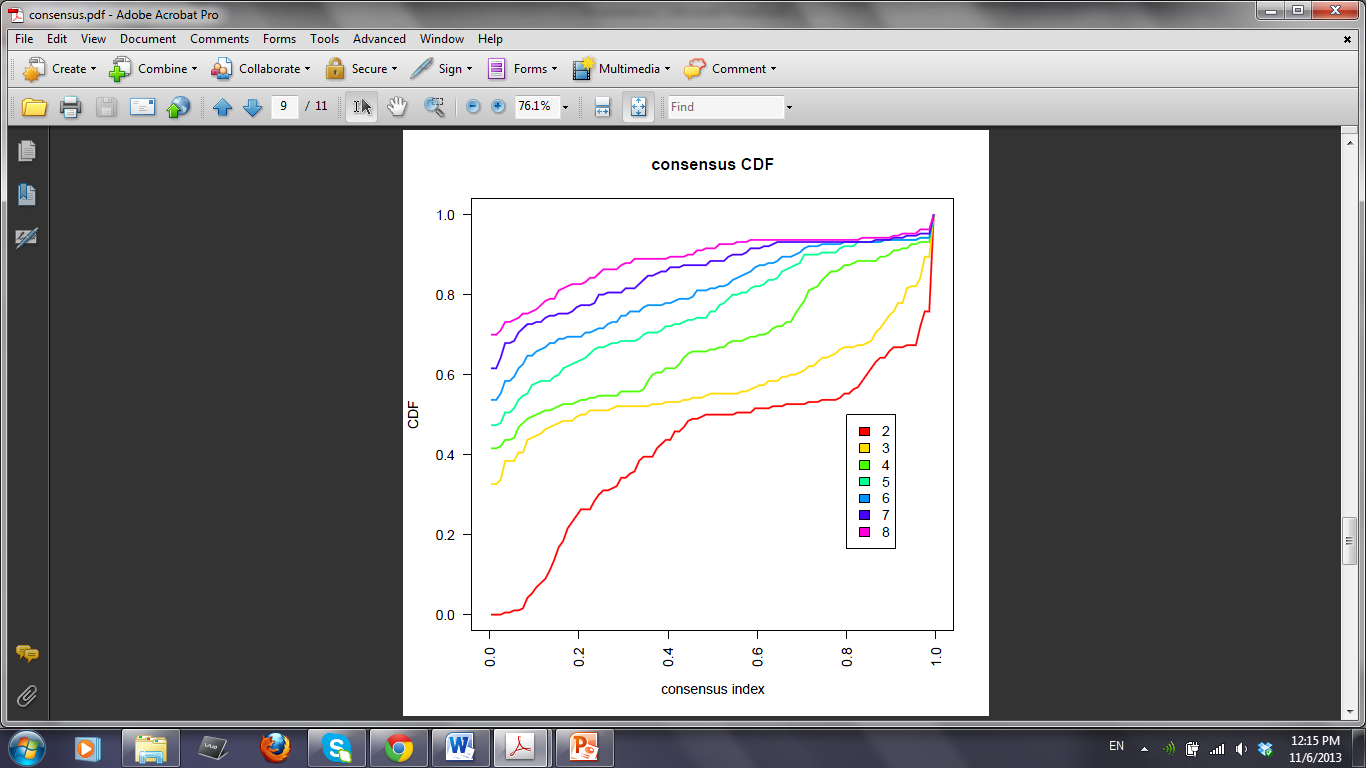


1. Area under the CDF curves comparing *K* and *K*-1 clusters


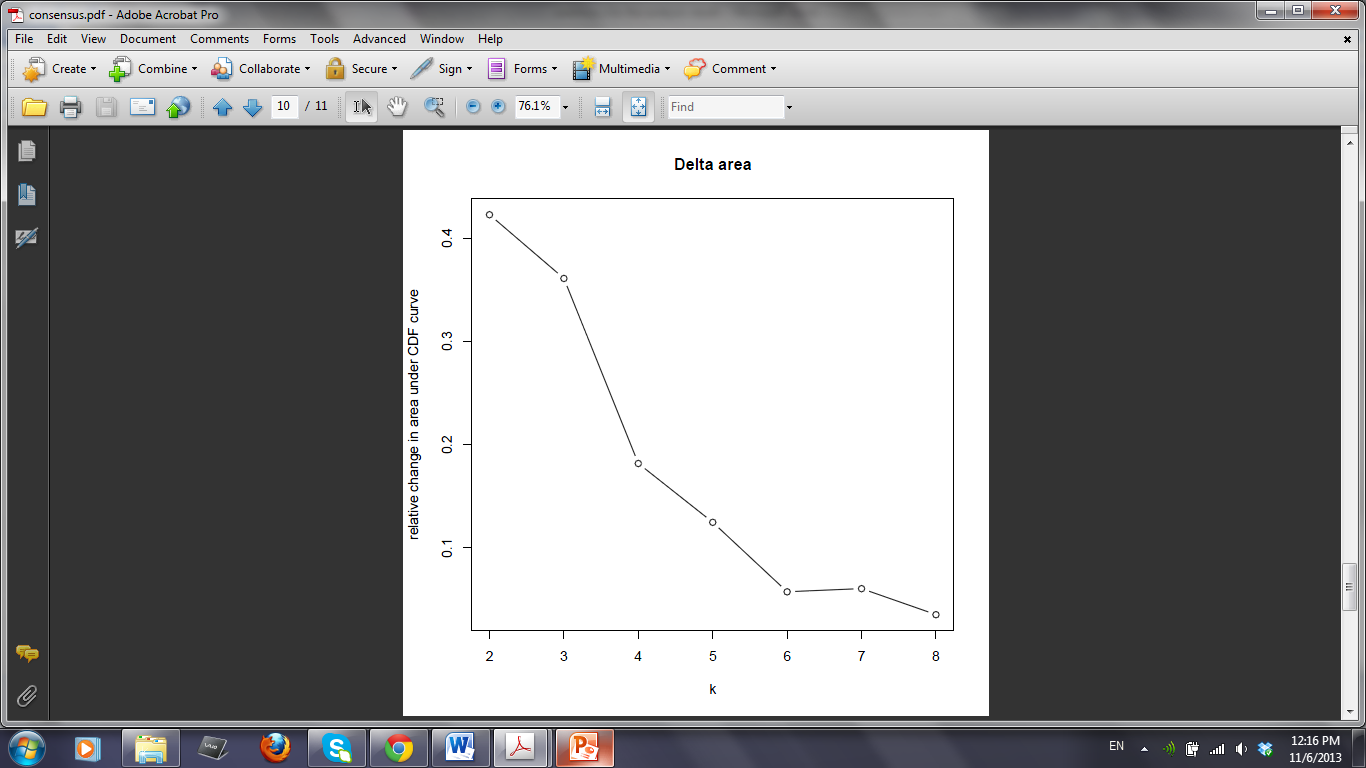


1. Cluster-consensus plot which calculates the mean of all pairwise consensus values between a cluster’s members at each *K*


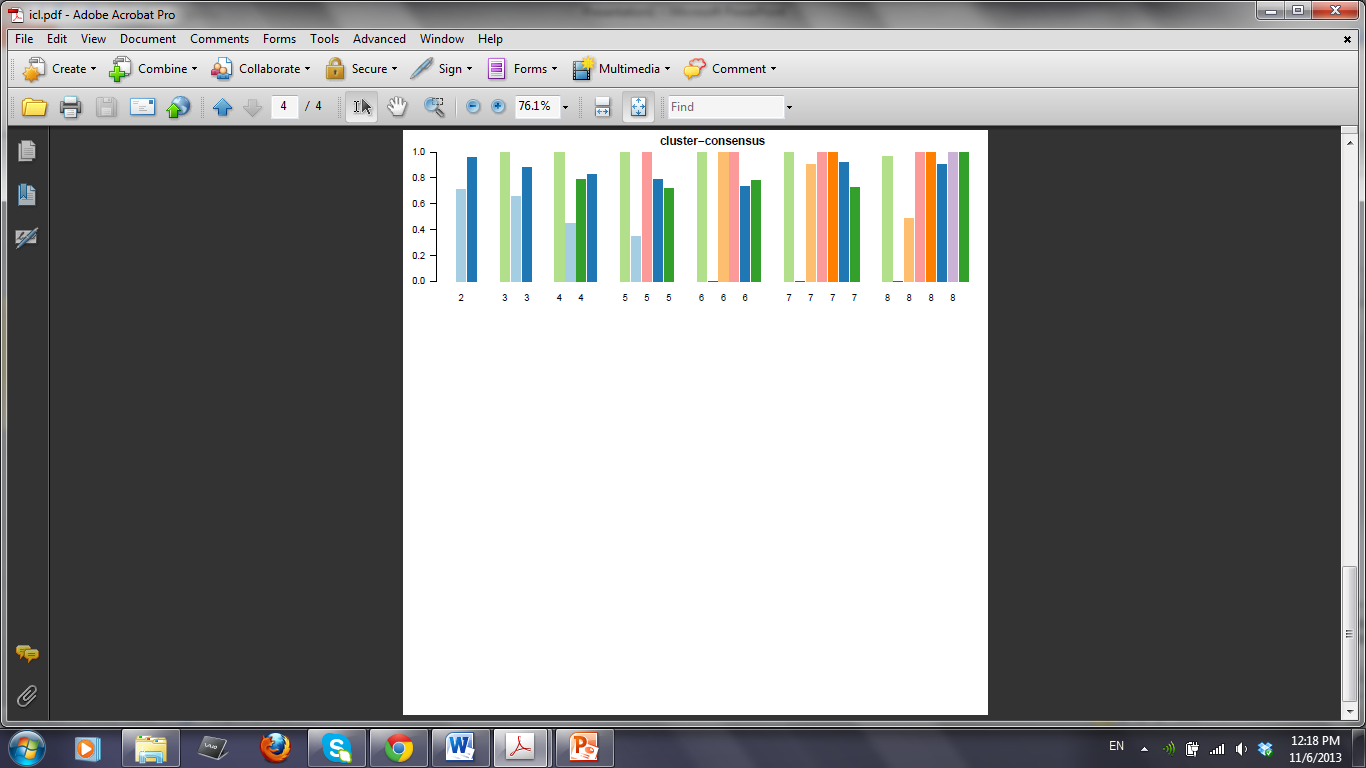


1. Cluster membership at *K*=5
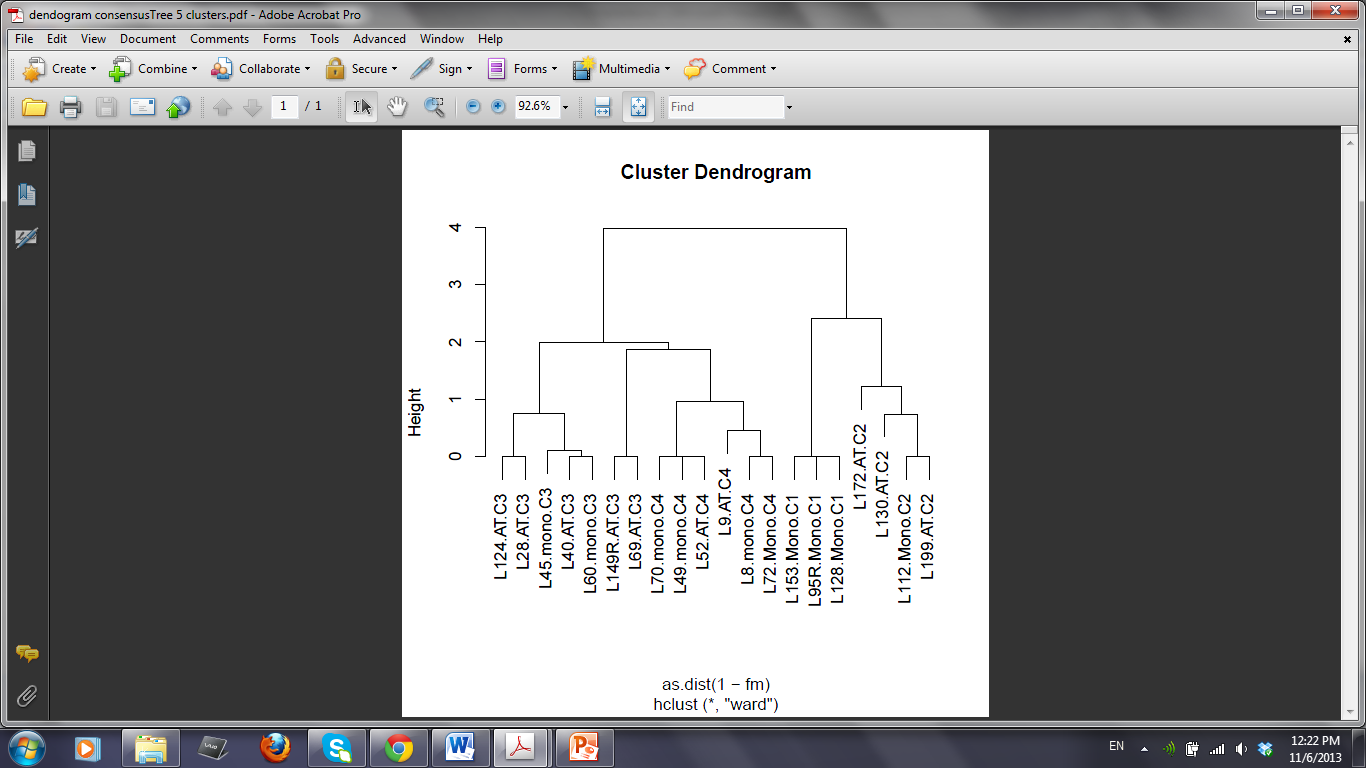

2. Cluster membership from original cluster structure indicated in figure 2


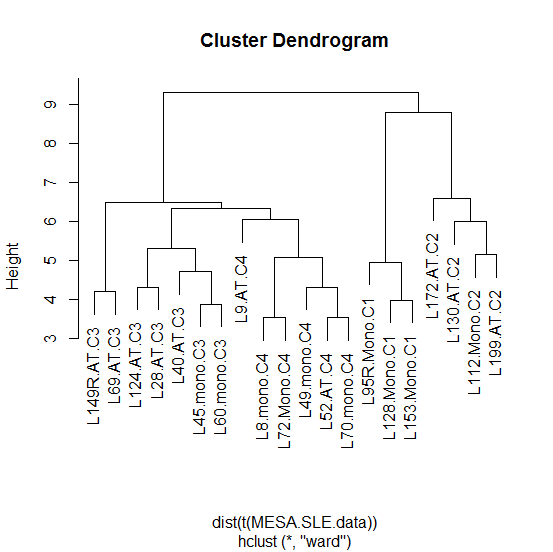


1. Tracking plot indicating cluster membership changes at K=5,6,7


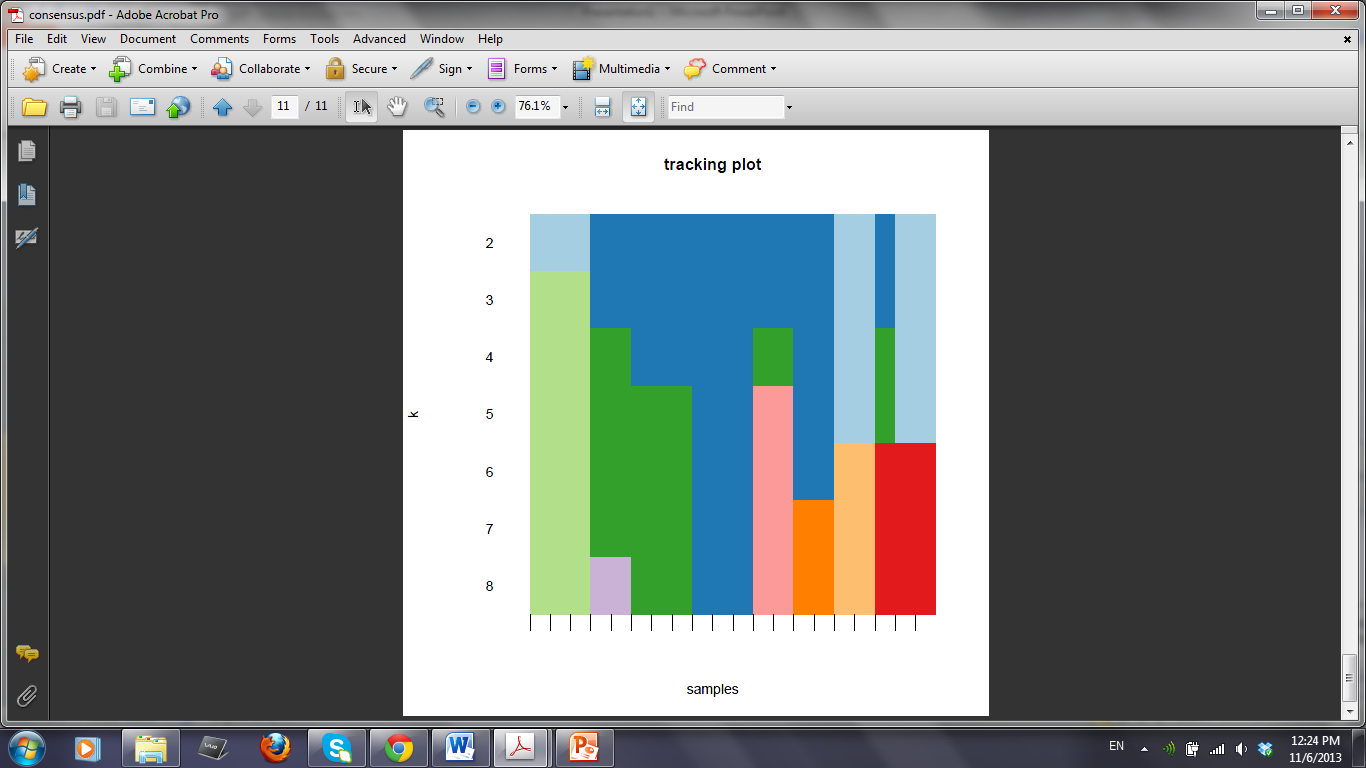


L153

L95

L128

L8

L72

L70

L49

L52AT

L40AT

L45

L60

L149AT

L69AT

L28AT

L124AT

L112

L199AT

L9AT

L130AT

L172AT
